# A Nonlinear Mixed Effects Approach for Modeling the Cell-To-Cell Variability of Mig1 Dynamics in Yeast

**DOI:** 10.1371/journal.pone.0124050

**Published:** 2015-04-20

**Authors:** Joachim Almquist, Loubna Bendrioua, Caroline Beck Adiels, Mattias Goksör, Stefan Hohmann, Mats Jirstrand

**Affiliations:** 1 Fraunhofer-Chalmers Centre, Chalmers Science Park, Göteborg, Sweden; 2 Systems and Synthetic Biology, Department of Chemical and Biological Engineering, Chalmers University of Technology, Göteborg, Sweden; 3 Department of Chemistry and Molecular Biology, University of Gothenburg, Göteborg, Sweden; 4 Department of Physics, University of Gothenburg, Göteborg, Sweden; Universitat Pompeu Fabra, SPAIN

## Abstract

The last decade has seen a rapid development of experimental techniques that allow data collection from individual cells. These techniques have enabled the discovery and characterization of variability within a population of genetically identical cells. Nonlinear mixed effects (NLME) modeling is an established framework for studying variability between individuals in a population, frequently used in pharmacokinetics and pharmacodynamics, but its potential for studies of cell-to-cell variability in molecular cell biology is yet to be exploited. Here we take advantage of this novel application of NLME modeling to study cell-to-cell variability in the dynamic behavior of the yeast transcription repressor Mig1. In particular, we investigate a recently discovered phenomenon where Mig1 during a short and transient period exits the nucleus when cells experience a shift from high to intermediate levels of extracellular glucose. A phenomenological model based on ordinary differential equations describing the transient dynamics of nuclear Mig1 is introduced, and according to the NLME methodology the parameters of this model are in turn modeled by a multivariate probability distribution. Using time-lapse microscopy data from nearly 200 cells, we estimate this parameter distribution according to the approach of maximizing the population likelihood. Based on the estimated distribution, parameter values for individual cells are furthermore characterized and the resulting Mig1 dynamics are compared to the single cell times-series data. The proposed NLME framework is also compared to the intuitive but limited standard two-stage (STS) approach. We demonstrate that the latter may overestimate variabilities by up to almost five fold. Finally, Monte Carlo simulations of the inferred population model are used to predict the distribution of key characteristics of the Mig1 transient response. We find that with decreasing levels of post-shift glucose, the transient response of Mig1 tend to be faster, more extended, and displays an increased cell-to-cell variability.

## Introduction

Cell biology data has traditionally been acquired by analyzing samples containing a large number of cells. However, data that has been produced by averaging the properties of individual cells may result in misleading interpretations of actual behaviors and underlying mechanisms [1–3]. Today, experimental methods are available that make it possible to measure certain quantities at the level of individual cells. These methods include techniques such as flow cytometry, fluorescence microscopy, and single cell transcriptomics, proteomics, and metabolomics. The development of experimental methods operating on single cells have enabled the study and characterization of cell-to-cell variability, adding a new dimension to the understanding of cell biology. For instance, flow cytometry has been used to study the population variability of the *GAL* regulatory network in yeast [4] and T cell activation [5]. This method produces snapshot data of the population at one or several time points. Each cell is only used for one single measurement, but the method can on the other hand be used to analyze a very large number of cells. For the generation of time-resolved data of the same particular cells, fluorescence microscopy of cells expressing proteins tagged with fluorescent proteins, e.g., GFP, has emerged as a powerful technique. Compared to the high-throughput capabilities of flow cytometry, time-laps imaging using fluorescence microscopy is typically carried out on a low- or medium-throughput scale. However, this data is substantially richer in information than snapshot data due to the temporal tracking of the same individual cells. Time-resolved data from single cells generated by the combination of microscopy and fluorescent proteins have been used in a large number of studies, including for instance investigations of nuclear accumulation of transcription factor activator ERK2 [1], golgi maturation in yeast [6], and stress-induced nuclear translocation of yeast kinase Hog1 [7] and transcription factors Crz1 [8] and Msn2 [9]. Although various cell-to-cell variability aspects of such data are increasingly being quantified and classified, the development of appropriate mathematical models and modeling approaches is still in its infancy. The need for suitable modeling approaches to describe the variability in dynamic behavior of cell populations has previously been pointed out by the authors of the present work [10], and by others [11], and research activities within this field are expected to increase.

Cell-to-cell variability between genetically identical cells, cultured under the same conditions, originates from the inherently stochastic nature of biochemical reactions. The sources of contribution to variability in gene expression can be separated into the effect of intrinsic noise on the actual reactions themselves, and extrinsic noise in the concentration of components participating in gene expression [12–14]. The latter concentrations are in turn ultimately also determined under the influence of intrinsic noise. Similarly, cell-to-cell variability may additionally originate from the intrinsic and extrinsic fluctuations in other parts of the cellular machinery, such as signalling pathways, and may further be impacted by small local differences in the external environment of individual cells. To mathematically model aspects of variability that are dominated by intrinsic noise, thus displaying noisy dynamics, stochastic approaches are required [2, 15, 16]. These typically involve the chemical master equation, or more commonly, approximations thereof. However, in many cases noise will establish itself as different expression-levels of various proteins, such as metabolic and signalling enzymes [5, 11, 14] and it is in fact often argued that such extrinsic noise is the dominant source of variability [12, 14, 17–19]. Cell-to-cell variability caused by different levels of protein expression can be described by deterministic models, where the values of parameters describing protein concentrations, enzymatic rate constants, etc., are distributed across the population. This approach was taken in a computational study on the behavior of protein kinase cascades [20]. Here, the authors explored the variability in signalling activity through simulations where enzyme concentrations were randomly sampled from log-normal distributions. In another study on the heterogeneous kinetics of ATK signalling [21], an ordinary differential equation model was fitted to average population data. The behavior of individual cells was then simulated by log-normal sampling of parameters representing enzyme concentrations. Still other examples can be found in modeling of the cell-to-cell variability of apoptosis signalling [18, 22, 23]. Importantly, in neither of these studies were the parameter distributions estimated using single-cell data.

Estimation of parameter distributions for models of heterogenous cell populations has previously addressed the special case of single cell snapshot data. This has been done using Bayesian approaches, for models with either deterministic [24] or stochastic [19] dynamics, and using maximum likelihood approaches for deterministic models [25]. Recently, Bayesian estimation methods for models with stochastic dynamics have also been customized for the case of time series measurements of the same single cells [26, 27]. In this work we extend on the approaches of deterministic single-cell dynamic modeling by incorporating parameter variability by means of so called nonlinear mixed effects (NLME) modeling, and estimating parameters from time series data using a maximum likelihood approach. NLME is a well-established and wide-spread approach to describe inter-individual variability between subjects of a population. It has a long history with numerous successful applications within various scientific fields [28], in particular including dynamical models in population pharmacokinetics and pharmacodynamics, but is sofar largely unexploited for addressing cell-to-cell variability in cell biology-oriented fields. An essential feature of the NLME framework is that all individuals of a population share the same model structure and that differences between subjects are due to different values of model parameters. Thus, the approach is suitable if it is reasonable to assume that the same mechanisms are controlling the behavior of different cells but quantitative details represented by parameter values may differ from one cell to another. This is implemented in the model by letting a subset of the parameters be described by a multivariate probability distribution, whose statistical properties are in turn parameterized by a set of additional parameters. Furthermore, as NLME facilitates the identification of parameters by considering the information from all individuals simultaneously, it is an especially appropriate modeling strategy when considering the often sparsely in time sampled data from single cells. We here apply NLME modeling in the novel context of single-cell data, using it to quantify the dynamic behavior of the yeast transcription factor Mig1.

Glucose and fructose are the most preferred carbon sources in *Saccharomyces cerevisiae* and the presence of any of these sugars activates the transcriptional repressor Mig1. This mechanism is referred to as glucose repression and involves genes required for the uptake and utilization of alternative carbon sources, gluconeogenic genes and the genes required for respiration [29]. A central role in glucose repression is played by the yeast AMP-activated protein kinase, Snf1 [30]. Snf1 is activated in response to glucose depletion by phosphorylation of the Thr210 residue within its activation loop [31]. This activation is promoted by any of the upstream activating kinases Sak1, Elm1 and Tos3 [32–34]. Snf1 phosphorylation is mainly antagonized by the activity of the Reg1-Glc7 protein phosphatase 1 (PP1) [35]. Active Snf1 phosphorylates the transcriptional repressor Mig1 promoting its dissociation from the co-repressor complex Ssn6 (Cyc8)-Tup1 and its nuclear export [36, 37]. Addition of glucose results in a rapid dephosphorylation of Snf1 and Mig1 and subsequently in nuclear accumulation of Mig1 [38, 39].

We recently published single-cell time-series data of Mig1 localization [39]. One of the interesting findings in that study was the behavior of Mig1 when glucose-grown cells experienced a shift in extracellular glucose from a high level (4%) to an intermediate level (1.5, 1.0, and 0.5%). In contrast to shifts to low concentrations of extracellular glucose, in response to which Mig1 persistently re-localized to the cytosol, shifts to intermediate levels of extracellular glucose caused Mig1 to first rapidly exit from the nucleus but then gradually return to its original nucleocytoplasmic distribution. Thus, it appears that the Snf1-Mig1 system can respond to a change in glucose concentration but depending on the absolute concentration level the system may perform some kind of adaptation. Such a transient response was an unexpected finding and the mechanism behind the apparent adaptation is unknown. In fact, considering a recent study involving 24 different mechanistic mathematical model variants [40], all based on up-to-date understanding of the Snf1-Mig1 system on the molecular level, none of the investigated models would be able to account for the transiently cytosolic Mig1. This can be realized by recognizing that in response to a change in extracellular glucose concentration, the accumulation of activations and inhibitions of every possible path for going from extracellular glucose to Mig1 will drive the Mig1 localization equilibrium in the same direction. Hence, none of the pathway combinations which were implemented in the different model variants are sufficient to explain the non-monotonic nature of the re-entry response. Furthermore, our single-cell time-series data clearly indicated that the extent and timing of the transient re-localization differed between individual cells. Although previous mathematical modeling efforts of the Snf1-Mig1 system have had access to data at the single cell level [40, 41], cell-to-cell variability has not yet been addressed.

In the present work, we set out to describe and quantify the previously reported nuclear exit and re-entry observations, focusing especially on the population variability aspect. Due to the lack of a mechanistically based hypothesis, a simple phenomenological model is developed. Using the NLME approach we are able to show that this model successfully captures the main characteristics of the transient behavior as it varies between individual cells. Importantly, we provide a model-based quantification of the cell-to-cell variability. This variability is reported in terms of estimated distributions of the model parameters. We show that there is a strong correlation between the two parameters determining the time-scales of nuclear exit and re-entry, respectively. This is an interesting finding as it offers a clue to the actual mechanism behind the exit and re-entry behavior. The NLME approach is furthermore compared to the simpler two-stage-approach [42]. While the latter appears to provide reasonable estimates of the median parameter values, it severely overestimates the population variability of the parameters and thus clearly demonstrates why NLME should be preferred. Finally, once parameter estimates have been obtained, the parameter variability of the population can be translated into variability of any model-derived property through Monte Carlo simulations. This type of analysis is used to investigate three key characteristics of Mig1 behavior, namely the median and variability of 1) the response time of Mig1 to a glucose shift, 2) the maximal response of nuclear exit, and 3) the duration of Mig1 cytosolic re-localization. A comparison with a simple non-model-based analysis suggests that these characteristics may not be immediately accessible from data alone. Hence, from a data quantification point of view the model, although only of phenomenological character, is crucial for extracting quantitative information about the process generating the data.

## Results

### Data description

This study relies on single cell data that we recently published [39]. In brief, these data were acquired from a Mig1-GFP expressing yeast strain using an experimental setup that is combining microfluidics, optical tweezers, fluorescence microscopy, and image processing. We study the scenario where glucose-grown cells are experiencing an instantaneous shift in extracellular glucose, going from 4% glucose to an intermediate level. In total, data from nearly 200 yeast cells, divided over four different data sets, are being used. The experiments are listed in Table 1.

**Table 1:** Experiments.

Exp Nr	Number of cells	From	To
1	56	4%	1.5%
2	46	4%	1.5%
3	46	4%	1%
4	46	4%	0.5%

List of experiments showing the experiment number, the number of cells used, and the levels of extracellular glucose.

**Fig 1:**
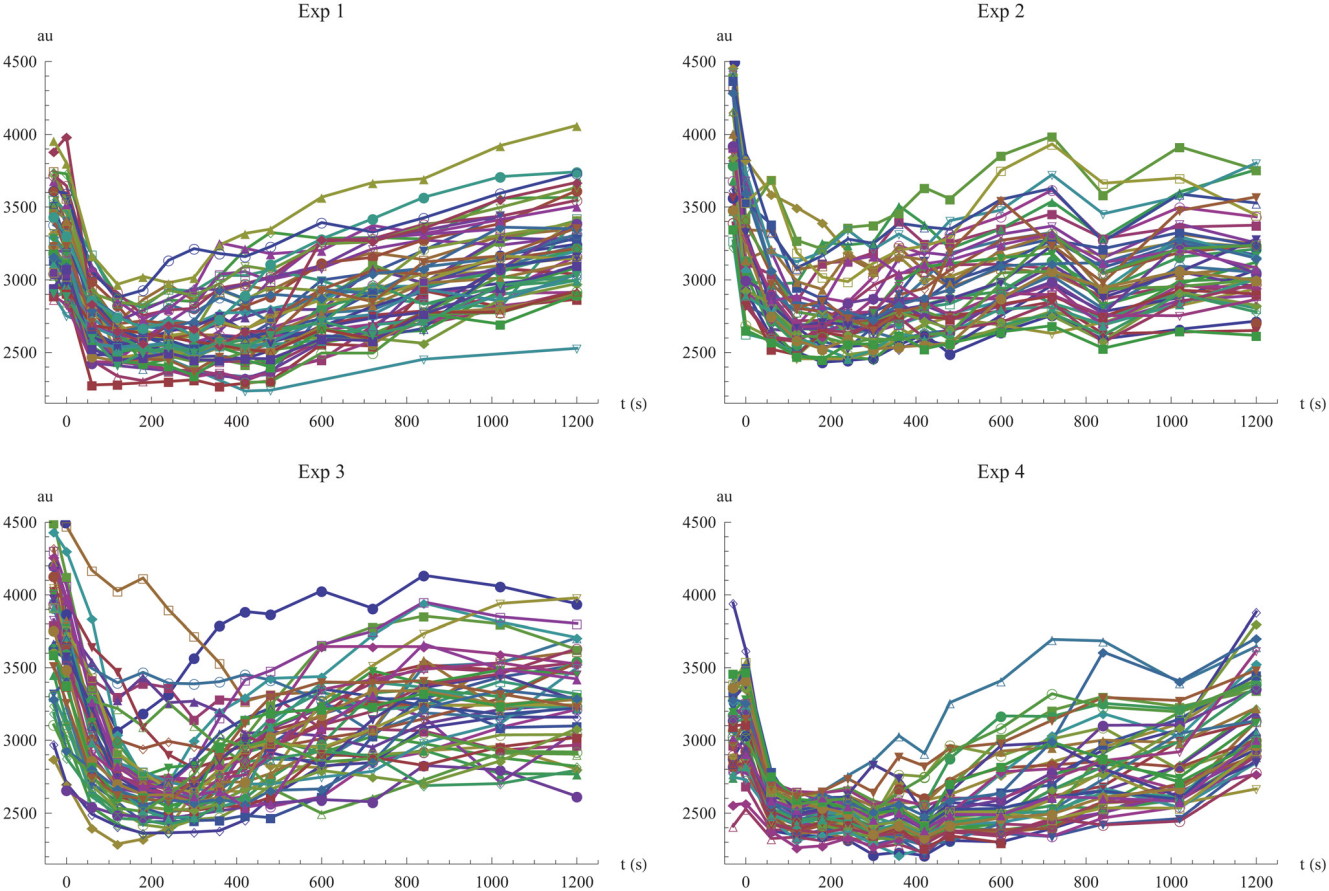
Visualization of all single cell data. Time-series data of fluorescent light intensity for nuclear Mig1 in single cells, shown for the four different experiments. At time zero, the extracellular glucose concentration is changed according to Table 1.

The data from experiments 1 to 4 is shown in Fig 1. The main feature of Mig1 behavior during these glucose exposure patterns is an initial rapid exit from the nucleus, followed by a slower re-entry, where Mig1 levels are readapting towards the baseline level prior to the glucose shift. Both the degree of Mig1 exiting the nucleus and the duration of the complete transient phase seem to increase with decreasing levels of extracellular glucose. All cells seem to share these characteristics but the baseline level of nuclear Mig1 and the timing and degree of exit and re-entry are varying between individual cells.

### Setting up a model

Signalling pathways are notoriously challenging to model because of the limited and uncertain knowledge of their components and the interactions between them [43–45]. Since state-of-the-art mechanistic modeling of the Snf1-Mig1 system does not support the transient Mig1 behavior described here [40], we instead aim for a phenomenological model that is as simple as possible, yet flexible enough to describe the Mig1 data. The simplicity of such a model is particularly important in our cases since there is only one measured species from which to calibrate the model, and since we are looking to infer not only parameters values but parameter distributions.

**Fig 2:**
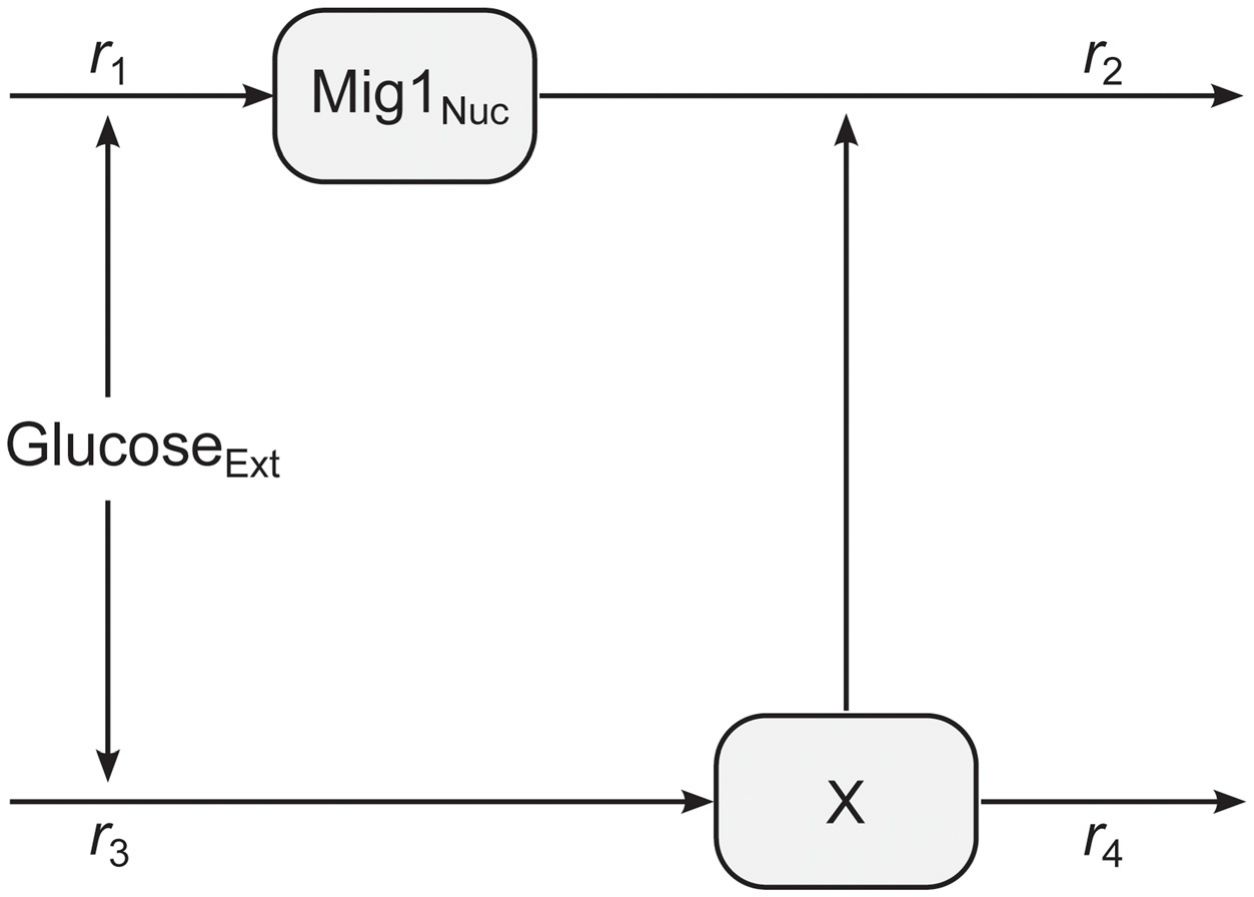
Illustration of the mathematical model. Extracellular glucose is controlling the rate of production of nuclear Mig1 and a hypothetical component X. The level of X in turn modulates the degradation of nuclear Mig1.

A minimal model of perfect adaptation was considered for modeling the dynamics at the single cell level. This model structure captures the main characteristics of the observed Mig1 behavior, while still providing some degree of interpretability with respect to the components and interactions of the model. The model is illustrated in Fig 2. It consists of two state variables, one representing the time-dependent concentration of Mig1(*t*) in the nucleus and one representing the time-dependent lumped effect, here denoted X(*t*), of one or several unknown components involved in the adaptation. Since we do not know the scaling factor between the observed fluorescent light intensity and the underlying actual concentration of Mig1 molecules, we chose to formulate the model in terms of the observed light intensities. The rate of accumulation of both state variables respond linearly to the level of extracellular glucose, Glu(*t*), which is treated as an experimentally controlled input to the system. Considering that the amounts of the involved components of the Snf1-Mig1 system are of the order 4 to 40 thousand molecules per cell [40], a deterministic model is assumed to be sufficient [46]. The mass balance equations for the state variables are defined by

$$\begin{array}{ccc} \frac{d\mathrm{Mig}1(t)}{dt} & = & r_{1}-r_{2}\\ \frac{dX(t)}{dt} & = & r_{3}-r_{4}, \end{array}$$

where the rates are defined as

$$\begin{array}{ccc} r_{1} & = & k_{1}\cdot \mathrm{Glu}\left(t\right)\\ r_{2} & = & k_{2}\cdot X\left(t\right)\cdot \mathrm{Mig}1\left(t\right)\\ r_{3} & = & k_{3}\cdot \mathrm{Glu}\left(t\right)\\ r_{4} & = & k_{4}\cdot X\left(t\right). \end{array}$$

The initial conditions are

$$\begin{array}{ccc} \mathrm{Mig}1(-30) & = & M_{\text{s}}\\ X(-30) & = & X_{\text{s}}, \end{array}$$

where we have chosen the initial time to -30 s with the convention that the input to the system is changed at time 0. The input to the system, the extracellular level of glucose, is

$$\begin{array}{ccc} \mathrm{Glu}(t) & = & 4-(4-g)\cdot H(t), \end{array}$$

where *H*(*t*) is the Heaviside step function and *g* is equal to either 1.5, 1, or 0.5 depending on the experiment. An observation of nuclear Mig1 at time *t*, *y*_*t*_, is modeled by introducing an additive error

$$\begin{array}{ccc} y_{t} & = & \text{Mig}1\left(t\right)+{\text{e}}_{t} \end{array}$$

where *e*_*t*_ ∼ 𝓝(0, *s*), with *s* denoting the variance of the measurement error. In a previous study of GFP-Mig1 [41] a moderate bleaching effect was identified from averaged single cell data. However, these experiments involved a substantially larger number of measurements (80 per cell and experiment compared to our 15) and the samples were likely bleached to a higher degree. We did not include the effect of fluorophore bleaching in our model, as the majority of cells displayed intensity levels which eventually returned close to the starting levels. In fact, a comparison of the intensities before the glucose shift and at 20 minutes showed that there was an average recovery level of 96%, a number that was determined despite the fact that all cells might not fully have completed their re-entry during the course of the experiment.

It is straightforward to show that the steady-state value of the response variable in the model is independent of the input signal [47]. In the context of Mig1-observations, this means that the model is limited to the experiments where the re-entry phenomena with perfect adaptation is manifested. To be able to describe Mig1 localization in response to a general perturbation in the glucose level, it is clear that some other kind of model would be necessary.

An important question in modeling arises when a model structure has been proposed but parameter values needs to be estimated from experimental observations; is there enough information in the data to uniquely determine the parameter values? If we in addition to Mig1(*t*) had been able to measure X(*t*), all parameters would have been *structurally identifiable* [48, 49]. However, when X(*t*) is not measured it turns out that the model is not identifiable, irrespective of the amount and quality of the data being used. If we let $\tilde{\text{X}}(t)=\alpha \text{X}(t)$, ${\tilde{k}}_{2}=k_{2}/\alpha$, and ${\tilde{k}}_{3}=\alpha \cdot k_{3}$, and multiply the differential equation for X(*t*) with *α*, the model equations can be written

$$\begin{array}{ccc} \frac{d\mathrm{Mig}1(t)}{dt} & = & k_{1}\cdot \mathrm{Glu}\left(t\right)-{\tilde{k}}_{2}\cdot \tilde{X}\left(t\right)\cdot \mathrm{Mig}1\left(t\right)\\ \frac{d\tilde{X}\left(t\right)}{dt} & = & {\tilde{k}}_{3}\cdot \mathrm{Glu}\left(t\right)-k_{4}\cdot \tilde{X}\left(t\right). \end{array}$$

This transformation leaves the measured state variable Mig1(*t*) unchanged, and in this sense results in an equivalent model. Thus, there is a redundancy in the dependence between X(*t*), *k*_2_, and *k*_3_ which prevents us from uniquely identifying these parts of the model. The crucial point, however, is that by choosing *α* to contain either the factor *k*_2_ or 1/*k*_3_, one of the parameters will cancel out and the transformed model will contain one parameter less. For instance, choosing *α* = *k*_4_/(*k*_3_·Glu(−30)), the parameter *k*_3_ will no longer appear in the equations and does not have to be estimated. This particular *α* also yields a very simple initial condition for $\tilde{\text{X}}(t)$. In this way we reduce the complexity of the original model but fully preserve its ability to describe the observed state variable Mig1(*t*). The fact that $\tilde{\text{X}}(t)$, ${\tilde{k}}_{2}$, and ${\tilde{k}}_{3}$ are different from the corresponding state variable and parameters of the original model is of no concern to us since they anyway represent aspects of a hypothesized process that is not defined on the molecular level, and hence there is no loss of interpretability. The model could have been reduced with respect to the parameter *k*_2_ instead, but since *k*_2_ will determine the turnover-timescale of Mig1(*t*) reduction with respect to *k*_3_ is more convenient.

For simplicity in notation, we will now drop the tildes and let the original names of variables and parameters refer to the reduced model. The equations defining the model in Fig 2 are

$$\begin{array}{ccc} \frac{d\mathrm{Mig}1(t)}{dt} & = & k_{1}\cdot \mathrm{Glu}\left(t\right)-k_{2}\cdot X\left(t\right)\cdot \mathrm{Mig}1\left(t\right)\\ \frac{dX(t)}{dt} & = & k_{4}\frac{\mathrm{Glu}(t)}{\mathrm{Glu}(-30)}-k_{4}\cdot X\left(t\right). \end{array}$$

Further model simplification can be achieved by acknowledging that the modeled system should be in steady-state at the beginning of each experiment. By assuming a steady-state at *t* = −30, we see that

$$\begin{array}{ccc} 0 & = & k_{1}\cdot \mathrm{Glu}\left(-30\right)-k_{2}\cdot X_{s}\cdot M_{s}\\ 0 & = & k_{4}-k_{4}\cdot X_{s}, \end{array}$$

and thus that the values of the model parameters are constrained by the initial values. From the second equality, we require that X_s_ = 1. We furthermore let the parameter *k*_1_ be a function of the other parameters and of the input according to

$$\begin{array}{c} k_{1}=\frac{k_{2}M_{s}}{\mathrm{Glu}(-30)}. \end{array}$$

This particular choice of reparameterization is motivated by the fact that the parameter *k*_2_ can be interpreted in terms of the turnover-timescale for Mig1(*t*) and M_s_ as the basal level of Mig1, making the resulting model most convenient.

We now turn to the population aspect of the mathematical model and how to account for the variability of the measured Mig1 dynamics in individual cells. In contrast to the non-random parameter values typically encountered in computational biology, variability between subjects is introduced by letting parameter values be described by probability distributions. Specifically, we chose to let the parameters of the dynamical model described above to be defined as the product of a so called fixed effect parameter, which involves no randomness, and a so called random effect parameter according to

$$\begin{array}{ccc} M_{\text{s}} & = & {\bar{M}}_{s}e^{\eta_{1}}\\ k_{2} & = & {\bar{k}}_{2}e^{\eta_{2}}\\ k_{4} & = & {\bar{k}}_{4}e^{\eta_{3}}. \end{array}$$

Here, the vector of random effect parameters, ***η*** = (*η*_1_, *η*_2_, *η*_3_), is normally distributed with zero mean and covariance matrix **Ω**. This means that the parameters M_s_, *k*_2_, and *k*_4_ are log-normally distributed. Their median values are determined by the parameters ${\bar{\text{M}}}_{\text{s}}$, ${\bar{k}}_{2}$, and ${\bar{k}}_{4}$, and their degree of variability is determined by **Ω**. The particular choice of a log-normal distribution is motivated by the universal appearance of this distribution in nature, ultimately originating from the fundamental laws of chemistry and physics [50, 51]. For instance, the concentrations of several mammalian signalling proteins have been shown to be log-normally distributed [5]. Since the proposed model is not a molecular-level mechanistic model, population variability of its parameters are meant to capture the aggregated effects of the underlying variability in all components relevant to Mig1 localization, ranging from proteins directly involved in Mig1 nucleocytoplasmic transport to proteins involved in sensing and signalling, etc.

### Estimating parameters

The experimental data described previously was used to estimate the parameters of the dynamical population model. This was done by maximizing the so called FOCE approximation of the population likelihood, using a gradient-based optimization scheme [52]. Three types of parameters were included in the parameter estimation:

- The fixed effect parameters of the model, ${\bar{\text{M}}}_{\text{s}}$, ${\bar{k}}_{2}$, and ${\bar{k}}_{4}$.
- The variance of the measurement noise, *s*.
- The parameters used to define the random effect covariance matrix, *ω*_11_, *ω*_12_, *ω*_13_, *ω*_22_, *ω*_23_, and *ω*_33_. Details of the parameterization of the random effect covariance matrix **Ω** are explained in the Methods section.

There are in a total 10 parameters to be estimated, collected in the vector

$$\begin{array}{c} \theta =({\bar{M}}_{\text{s}},{\bar{k}}_{2},{\bar{k}}_{4},s,\omega_{11},\omega_{12},\omega_{13},\omega_{22},\omega_{23},\omega_{33}). \end{array}$$

Each of the four experiments were considered separately, resulting in one set of estimates per experiment. The estimated values of the parameters for the different data sets are shown in Table 2. For each estimated parameter, its relative standard error (RSE) is shown within parenthesis. The estimate of the initial median level of nuclear Mig1, ${\bar{\text{M}}}_{\text{s}}$, is similar throughout the set of experiments. Experiments 1, 2 and 3 are similar with respect to the estimates of the parameters ${\bar{k}}_{2}$ and ${\bar{k}}_{4}$, while experiments 4 shows a slightly larger ${\bar{k}}_{2}$ and a ${\bar{k}}_{4}$ that is roughly double in size. The estimates of the measurement error variance differ for the different experiments. Moreover, it is clear that the parameters of the dynamical model are determined with high certainty, especially ${\bar{\text{M}}}_{\text{s}}$ which has a RSE of at most 2% in all of the four experiments. The values of the parameters used for constructing the covariance matrix for the random effect parameters are on the other hand somewhat more uncertain but the RSEs are in general still acceptable. One exception to this is RSE for *ω*_13_ in experiment 1. However, considering that RSE is a relative measure and that the estimate of this parameter value is close to zero, the absolute uncertainty is still low (standard error is 0.00128).

**Table 2:** Parameter estimates.

Parameter	Exp 1	Exp 2	Exp 3	Exp 4
${\bar{\text{M}}}_{\text{s}}$	3.27 × 10^3^ (1)	3.36 × 10^3^ (1)	3.64 × 10^3^ (2)	3.14 × 10^3^ (1)
${\bar{k}}_{2}$	0.00579 (4)	0.00473 (6)	0.00592 (9)	0.00815 (7)
${\bar{k}}_{4}$	0.00846 (4)	0.00971 (9)	0.00999 (9)	0.0229 (8)
*s*	8.73 × 10^3^ (6)	38.1 × 10^3^ (6)	20.8 × 10^3^ (6)	24.1 × 10^3^ (6)
*ω*_11_	0.0653 (11)	0.0712 (20)	0.0624 (12)	0.0228 (34)
*ω*_12_	0.0391 (28)	0.0447 (53)	0.0568 (22)	0.0691 (13)
*ω*_13_	47.5 × 10^−6^ (25598)	−0.0377 (49)	−0.0649 (24)	−0.0322 (46)
*ω*_22_	0.231 (12)	0.144 (44)	0.313 (13)	0.252 (15)
*ω*_23_	0.0398 (108)	0.193 (35)	0.526 (16)	0.281 (25)
*ω*_33_	0.255 (12)	0.439 (17)	0.567 (12)	0.432 (16)

Estimated parameter values and their corresponding relative standard error (expressed in percentage in the parenthesis), considering each of the four experiments separately.

Table 3 shows the covariance matrix for the random effect parameters, and the corresponding correlation matrix. For each matrix entry its RSE is shown within parenthesis. The correlation in population variability between *η*_1_ and *η*_2_ (associated with M_s_ and *k*_2_, respectively) is not very strong, and not showing a clear tendency across the experiments, but is on the other hand not very precisely estimated either. For experiments 2 to 4 there is a moderate negative correlation between *η*_1_ and *η*_3_ (associated with M_s_ and *k*_4_, respectively), and here correlation estimates are less uncertain. In these three experiments we also see that there is a substantial correlation between *η*_2_ and *η*_3_ (associated with *k*_2_ and *k*_4_, respectively), with the precision in the estimates being quite good. Experiment 1 on the other hand only suggest a weak correlation between *η*_2_ and *η*_3_, but may nevertheless be compatible with the other experiments since the estimated correlation is highly uncertain.

**Table 3:** Covariance and correlations matrices.

Exp Nr	**Ω**	Corr
1	$\left(\begin{array}{ccc} 0.0058\;(20) & 0.009\;(35) & 12.\times 10^{-6}\;(25684)\\ & 0.055\;(26) & 0.01\;(112)\\ & & 0.065\;(25) \end{array}\right)$	$\left(\begin{array}{ccc} 1 & 0.51\;(24) & 620.\times 10^{-6}\;(24736)\\ & 1 & 0.17\;(102)\\ & & 1 \end{array}\right)$
2	$\left(\begin{array}{ccc} 0.0085\;(27) & -840.\times 10^{-6}\;(735) & -0.017\;(58)\\ & 0.058\;(54) & 0.085\;(46)\\ & & 0.19\;(35) \end{array}\right)$	$\left(\begin{array}{ccc} 1 & -0.038\;(652) & -0.41\;(41)\\ & 1 & 0.8\;(22)\\ & & 1 \end{array}\right)$
3	$\left(\begin{array}{ccc} 0.011\;(22) & -0.016\;(66) & -0.037\;(31)\\ & 0.37\;(25) & 0.3\;(27)\\ & & 0.32\;(25) \end{array}\right)$	$\left(\begin{array}{ccc} 1 & -0.25\;(60) & -0.61\;(17)\\ & 1 & 0.86\;(6)\\ & & 1 \end{array}\right)$
4	$\left(\begin{array}{ccc} 0.0063\;(25) & 0.0083\;(75) & -0.014\;(55)\\ & 0.14\;(31) & 0.12\;(37)\\ & & 0.19\;(32) \end{array}\right)$	$\left(\begin{array}{ccc} 1 & 0.28\;(69) & -0.41\;(39)\\ & 1 & 0.74\;(14)\\ & & 1 \end{array}\right)$

Covariance and correlations matrices and their corresponding relative standard error (expressed in percentage in the parenthesis), considering each of the four experiments separately. The random effect parameters described by the first to the third row of these matrices, are associated with the fixed effect parameters ${\bar{\text{M}}}_{\text{s}}$, ${\bar{k}}_{2}$, and ${\bar{k}}_{4}$, respectively.

We additionally determined the maximum a posteriori estimates of the random effect parameters for each individual cell. These are the most likely values of ***η*** for an individual given the already estimated probability distribution for these parameters, and are also known as the empirical Bayes estimates (EBEs) [53]. To be able to trust further analysis involving the EBEs we determined the so called *η*-shrinkage, defined as the relative decrease in standard deviation of the EBEs compared to the standard deviation defined by the population estimate **Ω**. These values are shown in S1 Table. It is recommended that shrinkage should not be greater than 20 to 30% to avoid misleading conclusions in EBE-based diagnostics [53]. Although two of the percentages in experiments 2 are approaching such levels, the set of values as a whole should be considered feasible.

**Fig 3:**
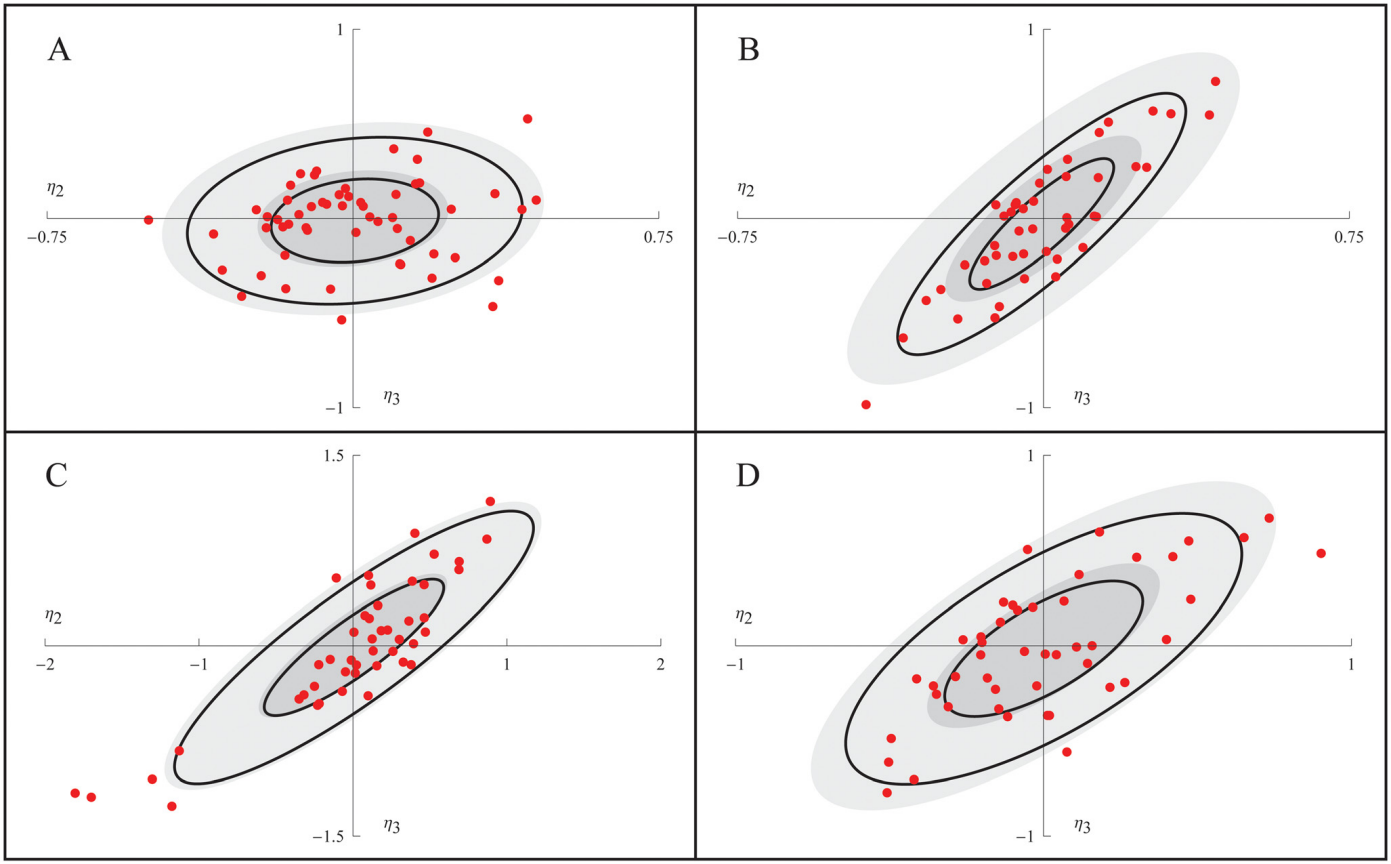
The distribution of maximum a posteriori *η*. For experiments 1 to 4 (A to D), the EBEs of *η*_2_ and *η*_3_ are shown as red points. The regions of one and two standard deviations of a normal distribution fitted to the EBEs, and the NLME population estimate of the distribution of *η*_2_ and *η*_3_, are shown as black and filled gray ellipses, respectively.

The EBEs were used to further investigate the correlation between *k*_2_ and *k*_4_. Fig 3 shows how the EBE values of the random effect parameters associated with ${\bar{k}}_{2}$ and ${\bar{k}}_{4}$, namely *η*_1_ and *η*_2_, are distributed in each of the four experiments. For each experiment, a normal distribution fitted to the EBE values is illustrated by two black ellipses, indicating the regions of one and two standard deviations. The distribution of *η*_2_ and *η*_3_ defined by the population estimate **Ω** is similarly illustrated by filled grey ellipses. This analysis confirmed the results displayed in Table 3. Again, there is only a slight correlation of the EBEs in experiments 1 (0.16), as shown in Fig 3A, but a pronounced correlation for the other three experiments (0.86, 0.87, 0.70), as shown in Fig 3B, 3C and 3D. The somewhat worse shrinkages of experiment 2 are also seen in Fig 3B as a difference between the filled and non-filled ellipses, respectively (although yielding very similar variances, please note that the black ellipses are based on fitting the EBEs to a normal distribution while the *η*-shrinkage is just based on the variance of the EBEs). In experiment 3, Fig 3C, five cells (with numbers #1, #2, #14, #26, and #29) stand out a bit from the others. Because of their comparatively more negative values of the random effect parameters, these cells have smaller effective values of *k*_2_ and *k*_4_ and should therefore display slower dynamics in response to the glucose shift. These cells may constitute a subgroup, but because of the relatively small sample size, and because of potential uncertainty in the EBEs of those cells, it is difficult to say with certainty. To make sure that these cells were viable and intact we went back to the raw images and inspected them manually. All cells looked normal although cell #29 appeared to be smaller and with a less developed nucleus.

### Comparing the inferred model to data

**Fig 4:**
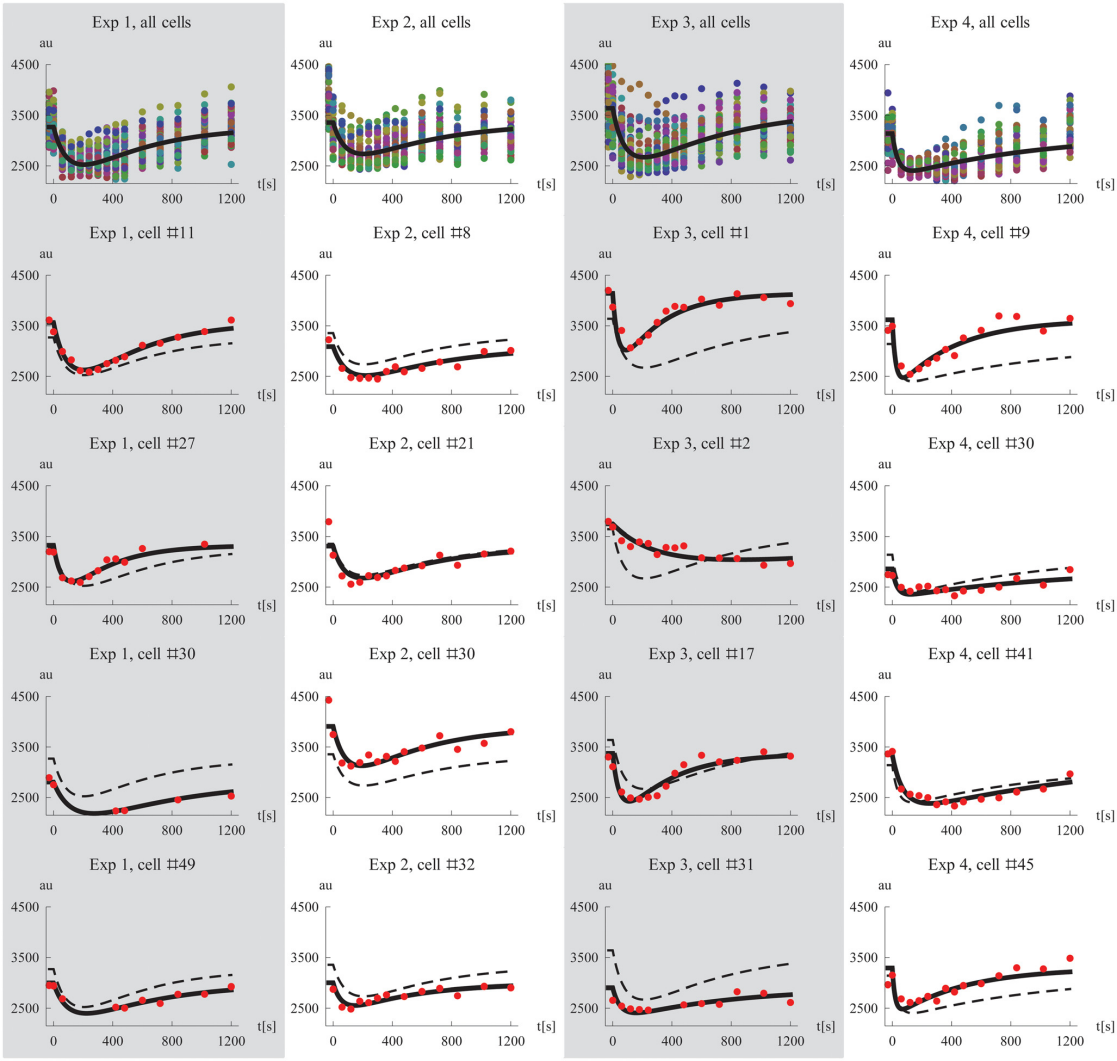
Model simulations and data. The first row show plots of all single cell data together with a simulation of a cell using the median parameters for each experiment, respectively. Rows two to five shows data and corresponding model simulations (derived using the EBEs) for a subset of all cells, exemplifying the fit on the individual level. The simulated median cell is shown in dashed for comparison. Columns one to four correspond to experiments 1 to 4.

The behavior of the model using the estimated parameter values was examined. We simulated the Mig1 dynamics of a typical cell by setting the random effect parameters to zero. For each of the four experiments, this simulation is shown together with the data from all cells in the first row of Fig 4. Additionally, we used the derived EBEs to simulate the model for specific cells and compared the results to the experimental observations. This was done for four representative cells per experiment and the results are shown in rows two to five in Fig 4. Plots of all individual cell data and model simulations for the four different experiments are shown in S1, S2, S3, and S4 Figs, respectively. Despite its simplicity the proposed model captures the different single cell Mig1 dynamics well, including cells with a “median response” (Exp 2 #21), high (Exp 2 #30) and low (Exp 3 #31) initial levels of Mig1, respectively, with fast (Exp 4 #9), and slow (Exp 4 #41) dynamics of the transient behavior, respectively, as well as cells with fewer data points (Exp 1, #30). We also note the unusually slow dynamics of cell #2 in experiment 3. This is one of the cells which we showed previously (Fig 3C) to have values of the EBEs that deviated from the others cells, and whose slower dynamics was already predicted at that point.

### Accounting for background fluorescence

The model was built under the assumption that the observed fluorescent light intensities are proportional to the actual concentration of Mig1. This assumption does not account for the presence of background fluorescence. To test whether the simplification of disregarding any background fluorescence is critical for the outcome of the analysis, we repeated the parameter estimation using the modified observational model

$$\begin{array}{ccc} y_{t} & = & b+\mathrm{Mig}1\left(t\right)+{\text{e}}_{t}, \end{array}$$

where *b* is a parameter to be estimated from data. The details of the parameter estimation are described in S1 Text, the results of the parameter estimation is shown in S2 Table and the corresponding random effect covariance and correlation matrices are shown in S3 Table, and plots of all individual cell data and model simulations for the four different experiments are shown in S5, S6, S7, and S8 Figs. In summary, changing the observation model to account for background fluorescence gave a marginally better fit to data but the parameter estimation suffered from issues with *practical identifiability* [54] and this model variant was therefore not considered further.

### Using all data sets simultaneously

**Fig 5:**
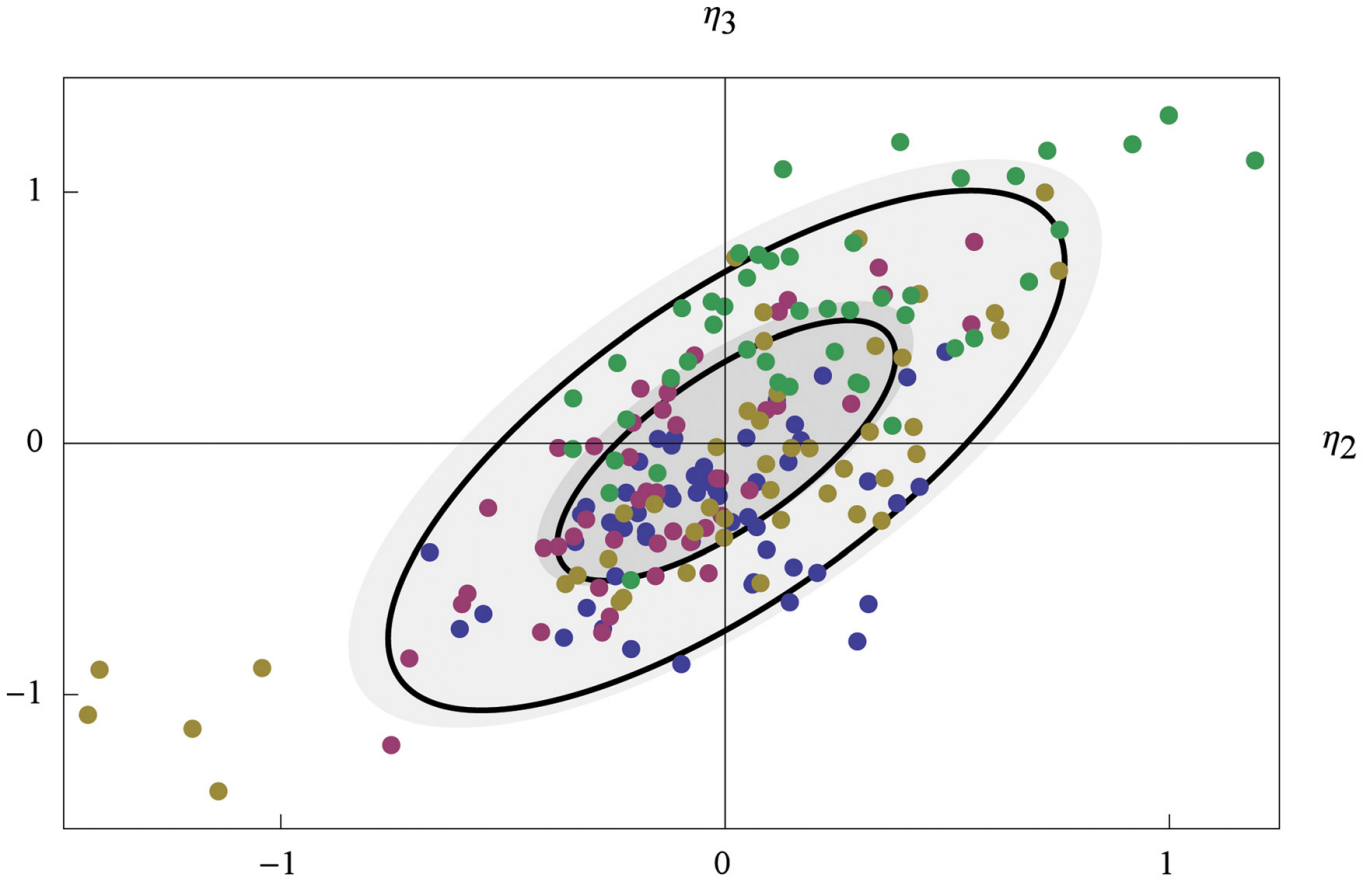
The distribution of EBEs of *η* for all cells in all experiments. The EBEs from individual cells are color-coded according to the experiments in which their data was produced using blue, pink, yellow, and green, for experiments 1 to 4, respectively.

Having estimated parameters successfully for each experiment separately, we decided to use all four data sets simultaneously for estimating the model parameters. The details of this analysis are described in S2 Text, the results of the parameter estimation is shown in S4 Table and the corresponding random effect covariance and correlation matrices are shown in S5 Table, and plots of all individual cell data and model simulations for the four different experiments are shown in S9, S10, S11, and S12 Figs. We then reinvestigated the distribution of the EBEs of the random parameters associated with ${\bar{k}}_{2}$ and ${\bar{k}}_{4}$, shown in Fig 5. As in Fig 3, a normal distribution fitted to the EBE values is illustrated by two black ellipses indicating the levels of one and two standard deviations, and the distribution of *η*_2_ and *η*_3_ defined by the population estimate **Ω** is similarly illustrated by filled grey ellipses. To separate the EBEs belonging to individual cells from the same experiment, we color-coded the dots for experiments 1 to 4 in blue, pink, yellow, and green, respectively. While the EBEs for experiments 1 to 3 display apparently similar distributions, though the five cells from experiment 3 still stand out, it is clear the cells from experiment 4 have consistently higher values of their random effect parameters, especially *η*_3_. Thus, even if the simulated Mig1 dynamics compare well with the single cell experimental observations, a model using the same parameter distributions $k_{2}={\bar{k}}_{2}e^{\eta_{2}}$ and $k_{4}={\bar{k}}_{4}e^{\eta_{3}}$ for all experiments is in some sense still misleading, and the results from the separate analysis should be considered more trustworthy.

### Comparing population parameter estimates to the statistics of single subject estimation

If every cell contains sufficient information to precisely estimate the parameters of the dynamical model, the parameters describing the population variability could simply be derived by fitting a parameterized distribution to the collection of all individual estimates. This straightforward approach to population modeling is known as the standard two-stage (STS) approach [42]. However, even moderate issues with identifiability for the parameter estimation of single cells may lead to biased estimates of population median parameters and overestimation of parameter variability. Being a much easier method to implement, and requiring substantially shorter times for computing the estimates, we decided to test whether the STS approach would be a feasible alternative to NLME. For each of the four experiments, the values of all random effect parameters were set to zero and the values of M_s_, *k*_2_, *k*_4_, and *s* were estimated for every cell. The resulting sets of parameter values were subsequently fitted to log-normal distributions. To avoid that extreme parameter estimates from uninformative single cell data sets had an unreasonably large impact on the estimated distributions, we repeated the analysis by removing single cell estimates that had at least one parameter value that differed more than 15 times from the median value of the set of individual estimates. This meant the exclusion of 2 cells from experiment 1, 3 cells from experiment 2, and 3 cells from experiment 4. No outliers were removed from experiment 3. The results of the comparison between STS and NLME is shown in Table 4, expressing the STS parameter estimates as percentages of the corresponding NLME estimates. The STS approach performed acceptably for estimating the median values of all experiments except for experiment 2 when all cells were used. When the outlier estimates had been removed it performs satisfactory for estimating median values in all experiments. The estimates for the measurement variance, *s*, were in all cases substantially lower. However, this parameter was not assigned to be distributed in the NLME approach, making the comparison more difficult. Importantly, with a few exceptions regarding the variance of M_s_, there is a clear overestimation in the variance of the model parameters, and this bias is in some cases considerable. It is obvious that a naive application of the STS approach, i.e., without screening for deviating values first, will give highly questionable estimates of the variability. Additionally we observed that even with a more careful use of the STS approach, variances may still be severely overestimated. For instance, the variance of *k*_2_ in experiment 2 is nearly five times larger when comparing the STS estimate to that of the NLME approach.

**Table 4:** Comparison of STS to NLME.

Parameter	Exp 1 All cells	Exp 1 3 cells removed	Exp 2 All cells	Exp 2 2 cells removed	Exp 3 All cells	Exp 4 All cells	Exp 4 2 cells removed
${\bar{\text{M}}}_{\text{s}}$	101	100	100	101	100	102	103
${\bar{k}}_{2}$	105	101	149	104	100	99	96
${\bar{k}}_{4}$	94	98	140	99	100	97	88
*s*	30	67	31	58	70	27	64
Var[M_s_]	101	97	123	107	109	412	394
Var[*k*_2_]	217	132	8797	490	129	510	177
Var[*k*_4_]	182	153	3329	206	142	839	217

The parameters estimates from the STS approach, either including all cells or removing cells with outlier estimates, expressed as percentages of the corresponding values derived from the NLME approach.

**Fig 6:**
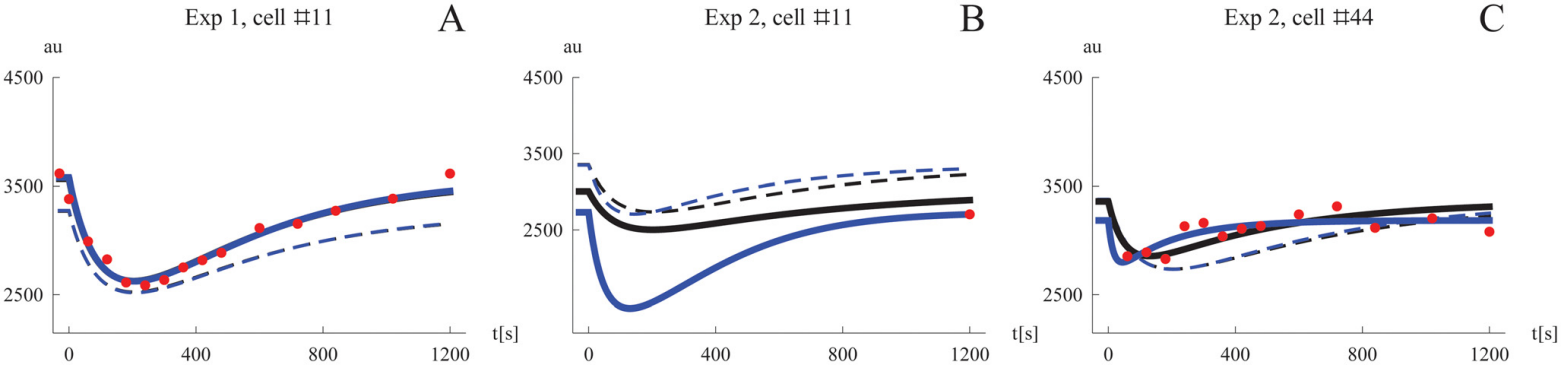
Comparing simulated Mig1 dynamics for individual cells using parameter from the STS and NLME approaches. Simulations with parameter values from the STS analysis are shown in blue, and in black for NLME. Simulations of typical cells are shown in dashed. A. An information-rich data set which by itself allows precise estimation of model parameters. The typical STS cell was simulated using the median parameters considering removal of outliers. B. The extreme case of an uninformative data set (only one data point). Here the STS approach may produce arbitrary parameter estimates which leads to questionable simulations as well as corrupting the population statistics of individual estimates. In this example the typical STS cell was simulated using the median parameters *without* considering removal of outliers, producing a different results compared to the typical NLME cell. C. A cell where the information content is too low for estimating all parameters with high precision. Model fits like this contribute to overestimation of parameter variability on the population level. The typical STS cell was simulated using the median parameters considering removal of outliers.

To illustrate why the STS approach gives different results than NLME three specific cells were examined more closely (Fig 6). Many cells contain an amount of information that is sufficient for the STS approach to produce similar estimates as the NLME at the single cell level. Fig 6A shows one such example where the simulations using the two different estimates practically look identical. In this example the NLME simulation used the value-triplet (3565, 0.00667, 0.00754) for the parameters (M_s_, *k*_2_, *k*_4_), and the STS simulation used the highly similar values (3578, 0.00668, 0.00738). When all cells are included in the analysis, a few rare time-series containing only one or two data points will be used. Fitting all model parameters to such data will produce completely arbitrary estimates due to lack of identifiability. This kind of scenario is shown in Fig 6B. Because the NLME approach is “borrowing” information (in form of the empirical prior) from the other cells when computing the estimate for a single cell, this simulation still resembles the median cell, while the STS simulation on the other hand produces a much more extreme behavior. We can also see that the simulated median cell of the population differs when the median parameters has been determined from all individual estimates. In this example the NLME simulation used the values (3007, 0.00442, 0.0104), while the STS used the very different values (2732, 0.0201, 0.00509). The inclusion of cells like these in the analysis is the reason why the STS approach where no estimates were discarded performed so badly. Fig 6B also shows that the dynamics of a typical cell derived from the STS approach without discarding outliers may differ to the typical cell of the NLME approach. As shown in Table 4 the STS approach can be improved by removing obvious outliers from the set of individual cell parameters. Although it is straightforward to remove parameter estimates from obviously noninformative data sets, e.g., time-series containing only a single data point, such preprocessing will to some extent be arbitrary. Consider for instance the single cell data in Fig 6C where the NLME and STS simulations used the values (3360, 0.00619, 0.0171), and (3185, 0.0151, 0.0557), respectively. There are 13 data points for this cell, yet it lacks the good identifiability properties from the example in Fig 6A. In such cases the STS approach tend to produce exaggerated, but not extreme, estimates, which contributes to bias and variability overestimation on the population level.

### Predicting the variability of the response activation time, amplitude, and duration

Having established an NLME model, it is possible to repeatedly simulate this model in order to determine the population-distribution of any property being described by the model. This was done to compute the population statistics of three quantitative measures of the transient Mig1 dynamics:

- **Response time**. The time it takes to reach the lowest concentration of nuclear Mig1 after a shift in extracellular glucose.
- **Amplitude**. The amplitude of the response measured in % below the baseline.
- **Duration**. The total time during which nuclear Mig1 remains below the level of half-maximum response.

**Fig 7:**
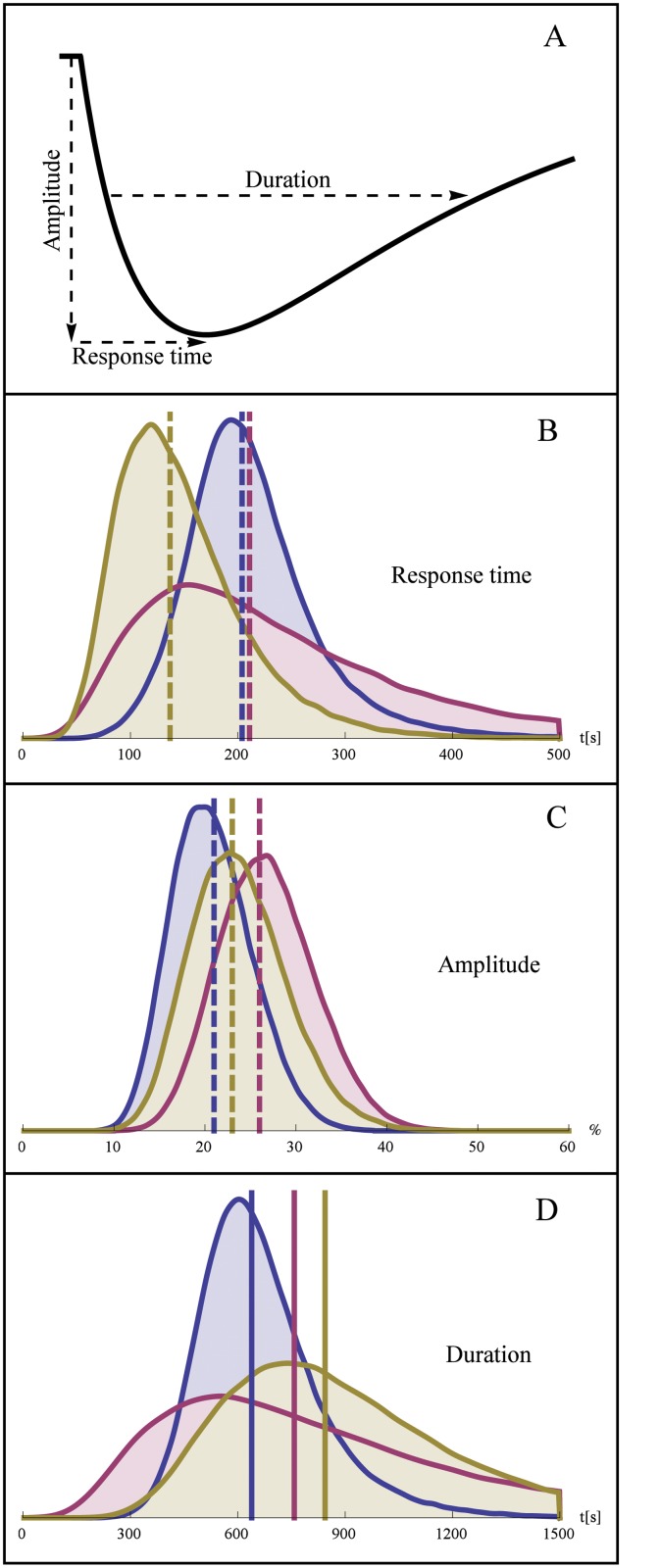
Distribution of the model-derived quantities response time, amplitude, and duration. A. Illustration of response time, (negative) response amplitude in % of baseline, and duration of half-maximal response. Distribution of activation (B), amplitude (C), and duration, for experiment 1 and 2 (blue), experiment 3 (pink), and experiment 4 (yellow). The typical cells (median response) are indicated by vertical dashed lines. Distributions of the model-derived quantities were determined from 100 000 Monte Carlo simulations per experiment.

These measures are illustrated in Fig 7A. According to the estimated population variability of the parameters M_s_, *k*_2_, and *k*_4_, we randomly created 100 000 in silico cells per experiment and simulated their Mig1 dynamics. The distributions and the typical values (medians) of the response time, amplitude, and duration are shown in Fig 7B, 7C and 7D, respectively. The typical values are also shown in Table 5. We observe that the simulated median response time is similar for concentrations of 1.5% and 1% glucose, respectively, but decreases markedly at 0.5%. Additionally, there is an increased variability of the response time for the intermediate concentration. The simulated amplitude of the Mig1 response exhibited quite small differences between the three conditions, both with respect to the median and the variability. A clear increase in median duration of the simulated response was observed as glucose concentrations decreases. The variability of the duration also increased with decreasing glucose levels. A similar behavior of the response duration was observed also when this quantity was defined by other levels than 50% of the maximum response (not showed).

**Table 5:** Typical values of response time, amplitude, and duration.

Quantity	Method	Exp 1 and 2 (1.5%)	Exp 3 (1%)	Exp 4 (0.5%)
Response time [s]	Model	204	211	137
Response time [s]	Simple analysis, experimental data	240	270	420
Response time [s]	Simple analysis, smoothed data	229	265	413
Amplitude [%]	Model	21	26	23
Amplitude [%]	Simple analysis, experimental data	23	29	23
Amplitude [%]	Simple analysis, smoothed data	23	28	22
Duration [s]	Model	639	758	844
Duration [s]	Simple analysis, experimental data	780	630	780
Duration [s]	Simple analysis, smoothed data	768	597	757

Typical values of time to full response, the amplitude of the response, and the duration of the response, obtained from the NLME model and from a simple non-model-based analysis using either the original or smoothed experimental data. The typical values of derived using the model were determined from 100 000 Monte Carlo simulations per experiment.

As a comparison to the model-based predictions of Mig1 dynamics, a simple non-model-based analysis was performed directly on the data and on dense data sets generated by smoothing and resampling the experimental data. The results from the simple analysis are shown together with the model-based predictions in Table 5. The simple analysis gave similar results for the amplitude, but did not identify an increased duration with decreasing extracellular glucose concentrations, and did furthermore suggest an opposite dependence of the typical response time on extracellular glucose concentrations when compared to the model-based predictions. Also, compared to the smooth distributions from the model-based analysis, the corresponding population histograms from the simple analysis were much less informative due to the limited number of cells and/or a binning based on the rather few discrete time points of the data, as shown for the simple analysis of the experimental data in S13 Fig.

## Discussion

State-of-the-art experimental techniques such as fluorescence microscopy allow time-resolved data to be collected from individual living cells. This development has provided researchers with tools enabling them to investigate various aspects of cell-to-cell variability in cell populations. The progress of single cell experimental methods requires a parallel advancement in the development of mathematical models for describing cell population heterogeneity. We propose that so called nonlinear mixed effects (NLME) models, a class of models that for example is used for modeling variability between individuals in pharmacological studies, also may be adopted for modeling cell-to-cell variability in molecular biology. The usefulness of this framework was demonstrated by applying a model of this type to study the localization dynamics of the yeast transcription factor Mig1. This protein is a key component in the regulation of carbon metabolism, responsible for repressing a larger number of genes in the presence of glucose. Using an NLME model we were able to quantify and simulate the cell-to-cell variability of yeast cells with respect to their behavior of Mig1. Comparing the proposed modeling methodology to a second more intuitive approach, we showed that the former is crucial in order to not overestimate the variability. An additional comparison of the NLME model to a simple non-model-based analysis indicated that modeling may be required for reliable interpretation of population data.

### Population model of Mig1 dynamics

We recently reported on a novel and unexpected aspect of Mig1 dynamics, namely the transient exit and subsequent nuclear re-entry of this protein in response to a shift from high to intermediate concentrations of extracellular glucose [39]. Similar transient responses followed by perfect adaption have been observed for other signalling proteins such as nuclear ERK2 in response to EGF levels [1], and the yeast kinase Hog1 in response to hyperosmotic shock [55]. Since the current understanding of the Snf1-Mig1 system does not provide a mechanistic basis for the apparent adaptation behavior, a simple phenomenological model of perfect adaptation was introduced to describe the observed Mig1 dynamics. The proposed model is a well known dynamical modeling motif and has previously been presented as one of the basic signal-response elements of regulatory networks [47]. Due to its simpler structure, the qualitative behavior of the model is limited to adaptation, with the parameter values controlling the the quantitative details of this behavior, and it can therefore not be used as a general-purpose model of Mig1 localization in response to extracellular glucose. To account for the observed cell-to-cell variability of Mig1 dynamics so called random effect parameters were introduced to the model. In contrast to most dynamical models used in computational biology, a subset of the model parameter values are now stochastic variables characterized by a distribution rather than scalar values. Although the model was not based on known molecular mechanisms for Mig1 regulation, it was successful in describing the experimental observations of Mig1 dynamics. It is however clear that even though such a phenomenological model can fit the data it may not provide the same fundamental insights of a mechanistically based model. Though, given the circumstances of limited knowledge of the mechanistic details of the Snf1-Mig1 system, we believe that the proposed model has an appropriate level of complexity, especially considering the population variability aspect, and that it may be a stepping stone towards future mechanistic models.

### Parameter estimates

The model performs well with similar median values of the time constants *k*_2_ and *k*_4_ both for 1.5% and 1% glucose, although with some variations in their variability. However, in experiment 4 the estimated time constant of the adaptation process, *k*_4_, was larger and a slightly larger value of *k*_2_ was obtained as well. The fact that other parameter values are required for this particular experiment can be seen as an indication that this level, 0.5% glucose, is close to a threshold in the behavior of the Snf1-Mig1 system. Indeed, this was also observed in experiments, where the transient behavior disappears for extracellular glucose levels below 0.5% [39]. It also suggests that to model all four experiments simultaneously, the linear response to glucose, as defined by the adaptation model, may not be sufficient.

Although the estimates of ${\bar{k}}_{2}$ and ${\bar{k}}_{4}$ appeared to be determined with good precision in the separate analysis, we decided to fix these parameters, including their distribution within the population, and estimate them from all experiments simultaneously. The resulting estimates were close to the average of the separate estimates. However, from the distribution of the EBEs (the maximum a posteriori estimates of the random parameters ***η***) it was obvious that the EBEs from the fourth experiment formed a separate cluster. This most likely violates the assumption that the random effect parameters from the different experiments are identically distributed and confirms what was already suspected based on the different and quite well-determined values of ${\bar{k}}_{2}$, and ${\bar{k}}_{4}$ in the separate analysis of experiment 4. Thus, the simultaneous analysis of all four experiments again suggests that the characteristics of Mig1 regulation is changing at a glucose level around 0.5%.

To account for background fluorescence we set up an alternative model of the measurement process. This did only result in a marginal improvement in the ability to explain the data, and since parameter estimation for this model appeared to experience problems with practical identifiability, it was not considered further. We want to stress that this does not mean that there is no background fluorescence, only that with the alternative model and the available data it appears unfeasible to estimate it. Finally, even though the results from this altered model should be interpreted cautiously due to the issues with parameter identifiability, we note that the model behavior was highly similar to the original model and that the correlation in population variability between *k*_2_ and *k*_4_ remained.

### Interpreting the model

Mig1 is continuously being transported in both directions across the nucleocytoplasmic interface and that its localization is dependent on the balance between these fluxes [39]. A change in Mig1 localization is thus due to a change in the balance between the rates of nuclear import and export. In light of this, the model can be interpreted as two counteracting mechanisms on Mig1 cellular localization: One quickly responding mechanism that promotes transport of Mig1 into the nucleus in response to an extracellular glucose signal (*r*_1_), and another delayed mechanism that counteracts the first one by promoting nuclear exit in response to glucose (the modulation of *r*_2_ by X). However, our present understanding of the signalling network controlling Mig1 activity does not include any mechanism that operates by favoring phosphorylation and cytosolic localization in response to the *presence* of glucose. Moreover, we observed a strong correlation in the cell-to-cell variability of *k*_2_ and *k*_4_, the parameters which determine the time scales of the two counteracting mechanisms. This means that if a mechanism of the second kind existed, it must be highly coordinated with the first one. As this might require a very precise orchestration in the expression of the hypothetically involved signalling components, we consider the explanation of counteracting mechanisms for the transient behavior even less likely. Taken together, it appears more plausible that the transient pattern is already present in the dynamics of an upstream pathway component that is controlling Mig1 localization according to the first type of mechanism. At least three candidate components may be considered for transmitting such a transient signal to Mig1:

- **Snf1**. This is a strong candidate since we know that on the averaged population level, Snf1 displays a temporal phosphorylation pattern that is similar to that of Mig1 localization [39]. However, the dynamics of Snf1 phosphorylation on the single cell level has not been investigated.
- **Glc7-Reg1**. Although mathematical modeling results and the lack of direct experimental evidence disfavor a direct regulation of Mig1 by Glc7-Reg1 [40], this scenario can not be ruled out. This phosphatase may alternatively transmit a transient signal indirectly via its effect on Snf1 phosphorylation.
- It has been observed that constitutively phosphorylated Snf1, as the result of overexpressing its upstream kinase Sak1, did not affect either Mig1 phosphorylation or its localization in the presence of glucose [40]. Based on this it was suggested that Snf1 activation is a necessary but not sufficient condition for mediating glucose de-repression, and that there must be a second glucose-regulated step directing Snf1 to Mig1. Such a mechanism may constitute the upstream source of the transient signal.

A combination of these scenarios would also be possible. Furthermore, the transient pattern need not emerge at the level of one of these components but could be present even further upstream, perhaps even in glycolysis itself which in a not fully understood manner generates the signal(s) for Snf1-Mig1 regulation. Further investigating the origin of the transient behavior, and the mechanisms behind its cell-to-cell variability, would be an interesting proposition for future single-cell studies.

A moderate negative correlation in the population variability of M_s_ and *k*_4_ was also found. This suggests a negative correlation between the levels of Mig1 and the timescale of the hypothesized adaptation process. This may very well be reasonable considering that molecular processes of the Snf1-Mig1 system which directly involve the Mig1 protein, such as phosphorylation and inter-compartment transport, may be subject to saturation effects. Thus, in cells where Mig1 levels are higher than average, the adaptation tends to be slower since a higher number of molecules has to be regulated by a capacity-limited system.

### Predicting the variability in response time, amplitude, and duration

Estimates of how parameters vary across the population can not only be analyzed as such, but they can also be used to derive the population variability of any system behavior described by the model. This can be achieved by Monte Carlo simulations using the inferred population model. Such model-based quantification is a powerful tool since it allows us to compute the cell-to-cell variability in aspects of Mig1 regulation which are not easily measurable directly from the time-series data. We used this approach to predict the population variability in three key determinants of the transient Mig1 response. From the results of this analysis (Fig 7) the following was concluded:

- The response time decreases as the level of the secondary glucose concentration decreases. Compared to the 1.5% level, the intermediate level (1%) additionally displays an increased cell-to-cell variability in the response time.
- The amplitude of the response, as determined relative to the baseline Mig1 level of each cell, appears to be largely independent of the glucose level, both with respect to its median value and with respect to its variability.
- The duration of the transient response is increased as the glucose level is decreased. Compared to the 1.5% level, there is also a clear increase in cell-to-cell variability of the duration.

To summarize, as the level of glucose after the shift is decreased, the transient Mig1 response tended to be faster and more extended, as well as showing an increased cell-to-cell variability in both of these two characteristics. Interestingly, we also note that all distributions of the investigated response characteristics appear to be log-normally shaped.

The model-based simulations of variability in response time, amplitude, and duration were also compared to a simple analysis based directly on the experimental data and on the corresponding smoothed and resampled data. Contrary to the model-based results, the simple data analysis did not identify an increasing duration of the response with decreasing extracellular glucose concentration, and did furthermore imply an increasing, rather than decreasing, response time with decreasing extracellular glucose concentration. Although such differences will depend on both the particular model used and on how the simple analysis is executed, the comparison suggests that a model-based approach may be more reliable for studying cell-to-cell variability in sparse or noisy data.

### NLME should be preferred to STS

We compared the results from NLME modeling to the more naive STS approach, which consists of performing parameter estimation on single cell data separately and subsequently fitting parameterized distributions to the resulting set of point estimates. Since the estimation of parameters for individual subjects do not rely on information from the rest of the population, the STS approach may tend to over-fit the data, potentially leading to biased estimates but even more commonly to overestimation of parameter variability [42]. Although the two methods provided comparable estimates of the median parameter values, the STS approach severely overestimated parameter variability. The results were particularly bad when estimates from some of the most sparse data sets were included. On the other hand, the NLME modeling approach was fully capable of handling these sparse data sets. In fact, even individuals with just a single observation were feasible and added information to the estimation. For the present study this meant that we did not have to discard any data, allowing us to use the available measurements optimally. Our data included up to 15 data points per individual cell. It is however realistic to assume that some single cell studies may involve substantially sparser sampling of certain quantities, creating an even stronger motivation in favor of the NLME approach compared to STS.

It must be recognized that sparseness in data, determined from counting the number of observations as such, may be a poor indicator for determining if the STS approach will be appropriate. Our comparison of the individual fit of the STS approach to the EBE-based estimate resulting from the NLME population estimation in Fig 6C illustrates this point. If this data set had been very rich in information the NLME-derived population prior would have had a minor impact on the EBE parameter estimate, and the resulting dynamics, and the two approaches would have produced similar results for this cell. Since this was not the case, it is clear that data sets which are not obviously sparse in the sense of containing very few observations (this data sets contained 13 data points) are not automatically suitable for the STS approach. The important question is rather whether the balance between information content in the data and the complexity of the model allows parameters to be estimated with high precision, considering the individual data sets in isolation. Thus, the advantage of NLME over STS is ultimately determined not only by the data sets at hand but also by the particular model being used. Another way of looking at the NLME approach compared to the STS approach would therefore be that the complexity of the model can be allowed to increase, beyond the point of practical identifiability in single subjects, as long as there is enough data on the population level.

Parameter estimates of individual single cell data have previously been performed in a model of the NF-*κ*B signalling pathway [56]. Here, 6 parameters were estimated for 20 different cells using 15 data points per cell, and the authors noted that some of the parameters were estimated with a quite high uncertainty. Had parameter distributions been fitted to these single cell estimates, the risk of overestimating parameter variability would probably had been high. In another mathematical modeling study of cell-to-cell variability [23], parameter estimation at the single cell level was performed by complementing the single cell time lapse data with other types data, with the purpose of increasing parameter identifiability.

### The need for population modeling frameworks

The idea of applying hierarchical modeling, such as NLME, to longitudinal population data acquired at the level of single cells has previously been acknowledged and outlined by the authors of this work [10]. Since then, initial efforts towards single cell modeling using the NLME approach have in fact been considered in a few cases [57, 58], but the full potential of the approach has yet to be realized. The present study is to our knowledge the first one to combine, and in detail cover, aspects of NLME modeling such as uncertainty of estimates, investigation of EBEs and comparison of simulations to single cell data, and using an estimated model for prediction. Also, this is the first study in which NLME has been applied not only with a focus on its technical aspects but also with an ambition to advance the understanding of cell biology.

In parallel with the developments within NLME modeling, single cell time series data have recently also been approached using hierarchical Bayesian methods [26, 27]. In addition to extrinsic variability these efforts also considered intrinsic noise. Although a deterministic approach seems to describe the single cell Mig1 data studied here quite well, an extension of the NLME approach to also cover uncertainty in the dynamics would be interesting. One way of achieving this would be to replace the ordinary differential equation by so called stochastic differential equations (SDEs). The combination of NLME and SDE has previously been considered in pharmcokinetics and pharmcodynamics [59–61]. Not only would this allow intrinsic noise to be addressed within the NLME framework, but the SDEs could also be used to account for miss-specification of the deterministic parts of a model. Applying dynamical modeling with SDEs towards this end has previously proven useful for guiding the process of model development [62]. This strategy may be especially rewarding for modeling of signalling transduction pathways, as these systems typically suffer from limitations and uncertainty in the information needed for setting up models.

The need for modeling frameworks that are able to address single cell data is perhaps most clearly demonstrated by a growing number of studies in which such data was collected but then averaged during the computational analysis [41, 63]. We predict that hierarchical modeling frameworks such as NLME modeling will become even more important as single cell experimental methods continue to develop, and as the biological questions will involve the single cell perspective to a larger extent. In the future, dynamical modeling of non-genetic cell-to-cell variability may not only be relevant for basic research but also become an important ingredient in various applied fields of life science such as quantitative pharmacology [11] and industrial biotechnology [64]. As previously pointed out [58], an intriguing future prospect of single cell NLME modeling is the inclusion of so called covariates in the model. Covariates are known individual-specific variables which are used to account for predictable sources of the variability. In pharmacokinetic modeling, which frequently uses the NLME approach, covariates may for instance include weight, age, and sex. In the context of single cell modeling, the addition of covariates to the model could be used to incorporate cell-specific information such as size, shape, or age, in addition to the time-series data. Another important challenge for system identification from population data is the development of methods that can handle the combination of measurements at the single cell level with the traditional type of data produced from averaging over many cells.

## Methods

The yeast strains, experimental setup, and imaging and image analysis, have been described previously [39].

### Parameter estimation for NLME models

NLME models are often used in situations where sparse time-series data is collected from a population of individuals subject to inter-individual variability. These models contain both so called fixed effect parameters, being non-random, and so called random effect parameters, which are determined by some statistical model. Given a set of population data and a NLME model, the fixed effect parameters can be estimated according to the maximum likelihood approach. The likelihood subject to maximization is the so called population likelihood. This is a special kind of likelihood that has been marginalized with respect to all random effect parameters, and that is taking the observations from all individuals of the population into account. We now state the general form of a NLME model, the population likelihood, and its approximation by the so called FOCE method.

Consider a population of *N* subjects and let the *i*th individual be described by the dynamical system

$$\begin{array}{ccc} \frac{dx_{i}\left(t\right)}{dt} & = & f(x_{i}\left(t\right),u_{i}\left(t\right),Z_{i},\theta ,\eta_{i},t)\\ x_{i}\left(t_{0}\right) & = & x_{0i}\left(u_{i}\left(t_{0}\right),Z_{i},\theta ,\eta_{i}\right), \end{array}$$

where **u**_*i*_(*t*) is a time dependent input function, **Z**_*i*_ a set of covariates, ***θ*** a set of fixed effects parameters, and ***η***_*i*_ a set of random effect parameters which are multivariate normally distributed with zero mean and covariance **Ω**. The covariance matrix **Ω** is in general unknown and will therefore typically contain parameters subject to estimation. These parameters will for convenience of notation be included in the fixed effect parameter vector ***θ***. A discrete-time observation model for the *j*th observation of the *i*th individual at time *t*_*ij*_ is defined by

$$\begin{array}{c} y_{ij}=h\left(x_{ij},u_{ij},t_{ij},Z_{i},\theta ,\eta_{i}\right)+e_{ij}, \end{array}$$

where

$$\begin{array}{c} e_{ij}∼N\left(0,R_{ij}\left(x_{ij},u_{ij},t_{ij},Z_{i},\theta ,\eta_{i}\right)\right), \end{array}$$

and where the index notation *ij* is used as a short form for denoting the *i*th individual at the *j*th observation. Furthermore, we let the expected value of the discrete-time observation model be denoted by

$$\begin{array}{c} {\hat{y}}_{ij}=E\left[y_{ij}\right]. \end{array}$$

Given a set of experimental observations, **d**_*ij*_, for the individuals *i* = 1,…, *N* at time points *j* = 1,…, *n*_*i*_, we define the residuals

$$\begin{array}{c} \epsilon_{ij}=d_{ij}-{\hat{y}}_{ij}, \end{array}$$

and write the population likelihood

$$\begin{array}{c} L\left(\theta \right)=\prod_{i=1}^{N}\int p_{1}\left(d_{i}|\theta ,\eta_{i}\right)p_{2}\left(\eta_{i}|\theta \right)\;d\eta_{i}, \end{array}$$ (1)

where

$$\begin{array}{c} p_{1}\left(d_{i}|\theta ,\eta_{i}\right)=\prod_{j=1}^{n_{i}}\frac{\exp(-\frac{1}{2}\epsilon_{ij}^{T}R_{ij}^{-1}\epsilon_{ij})}{\sqrt{\det(2\pi R_{ij})}} \end{array}$$

and

$$\begin{array}{c} p_{2}\left(\eta_{i}|\theta \right)=\frac{\exp(-\frac{1}{2}\eta_{i}^{T}\Omega^{-1}\eta_{i})}{\sqrt{\det(2\pi \Omega )}}. \end{array}$$

The marginalization with respect to ***η***_*i*_ in Eq 1 does not have a closed form solution. By writing Eq 1 on the form

$$\begin{array}{c} L\left(\theta \right)=\prod_{i=1}^{N}\int \exp\left(l_{i}\right)\;d\eta_{i}, \end{array}$$

where the individual joint log-likelihoods are

$$\begin{array}{ccc} l_{i} & = & -\frac{1}{2}\sum_{j=1}^{n_{i}}(\epsilon_{ij}^{T}R_{ij}^{-1}\epsilon_{ij}+\log\det\left(2\pi R_{ij}\right))\\ & & -\frac{1}{2}\eta_{i}^{T}\Omega^{-1}\eta_{i}-\frac{1}{2}\log\det\left(2\pi \Omega \right), \end{array}$$

a closed form solution can be obtained by approximating the function *l*_*i*_ with a second order Taylor expansion with respect to ***η***_*i*_. This is the well-known Laplacian approximation. Furthermore, we let the point around which the Taylor expansion is done to be conditioned on the ***η***_*i*_ maximizing *l*_*i*_, here denoted by $\eta_{i}^{*}$, and we approximate the Hessian used for the expansion with first order terms only. Thus, the approximate population likelihood *L*_*a*_ becomes

$$\begin{array}{c} L\left(\theta \right)\approx L_{a}\left(\theta \right)=\prod_{i=1}^{N}(\exp\left(l_{i}\left(\eta_{i}^{*}\right)\right)\det{\left[\frac{-\Delta l_{i}\left(\eta_{i}^{*}\right)}{2\pi }\right]}^{-\frac{1}{2}}). \end{array}$$

where

$$\begin{array}{c} \Delta l_{i}\left(\eta_{i}^{*}\right)\approx -\sum_{j=1}^{n_{i}}\nabla \epsilon_{ij}^{T}R_{ij}^{-1}\nabla \epsilon_{ij}-\Omega^{-1}, \end{array}$$

and

$$\begin{array}{c} \nabla \epsilon_{ij}={\frac{\partial \epsilon_{ij}}{\partial \eta_{i}}|}_{\eta_{i}=\eta_{i}^{*}}. \end{array}$$

This variant of the Laplacian approximation of the population likelihood is known as the first order conditional estimation (FOCE) method [65].

The maximum likelihood estimate of ***θ*** is obtained by maximizing the approximate population likelihood *L*_*a*_(***θ***). The parameters being estimated are all parameter included in ***θ***, namely the fixed effect parameters of the dynamical model, including the fixed effect parameters of the observational model, and any parameters appearing in the random effect covariance matrix **Ω**. The optimization problem resulting from the desire to maximize *L*_*a*_ with respect to ***θ*** was solved using the BFGS method [66]. Note that every evaluation of *L*_*a*_ requires the determination of $\eta_{i}^{*}$ for all individuals due to the conditional nature of the FOCE approximation. Thus, the optimization of *L*_*a*_ with respect to ***θ*** involves a nested optimization of *l*_*i*_ with respect to ***η***_*i*_ for every individual, making the parameter estimation a challenging problem. An exhaustive account of how the gradient-based optimization was performed for the FOCE approximation of the population likelihood can be found in [52].

Since the approximate population likelihood involves a marginalization over the random effect parameters ***η***_*i*_, these are not explicitly estimated. However, once the estimate of ***θ*** has been obtained, the maximum a posteriori estimates of the random effect parameters for each individual cell (referred to as empirical Bayes estimates in the results section) can be determined. These are in fact equivalent to $\eta_{i}^{*}$, meaning that they are already provided as an indirect effect of the final evaluation of *L*_*a*_.

### Parameterization of the random effect covariance matrix

The elements of the random effect covariance matrix **Ω** cannot be chosen independently from one another. To ensure that **Ω** will be positive semi-definite and symmetric, and thus a covariance matrix, it is decomposed into **Ω** = **U** **U** ^*T*^, where **U** is an upper triangular matrix which can be parameterized according to

$$\begin{array}{c} U=(\begin{array}{ccc} \omega_{11} & \omega_{12} & \omega_{13}\\ & \omega_{22} & \omega_{23}\\ & & \omega_{33} \end{array}). \end{array}$$

Such decomposition is only unique if **Ω** is strictly positive definite and if the diagonal elements of **U** are positive. The sought-after covariance matrix can for practical purposes always be considered positive-definite, and since we are not interested in **U** as such we do not care about the signs of its diagonal entries. With the parameterization above, **Ω** becomes

$$\begin{array}{c} \Omega =(\begin{array}{ccc} \omega_{11}^{2}+\omega_{12}^{2}+\omega_{13}^{2} & \omega_{12}\omega_{22}+\omega_{13}\omega_{23} & \omega_{13}\omega_{33}\\ \omega_{12}\omega_{22}+\omega_{13}\omega_{23} & \omega_{22}^{2}+\omega_{23}^{2} & \omega_{23}\omega_{33}\\ \omega_{13}\omega_{33} & \omega_{23}\omega_{33} & \omega_{33}^{2} \end{array}). \end{array}$$

### Uncertainty of parameter estimates

The uncertainty of parameter estimates are reported as relative standard errors. The relative standard error is computed by taking the absolute value of the ratio between the standard error of the parameter estimate to the estimated value, expressed in percentage. Parameter standard errors are obtained by taking the square root of the diagonal elements of the inverse of the negative Hessian, calculated at the points of the estimated parameter values. Since the uncertainties in the entries of **Ω** (and the corresponding correlation matrix) depend on various combination of parameter uncertainties, they were determined by computing the RSE from a large number of sampled covariance and correlation matrices.

We note that measures of confidence based on the exact likelihood, such as likelihood profiling, typically are superior to the results from asymptotic theory. Although preferable, such methods are too time-consuming for the large NLME problems considered here.

### Starting values for the optimization algorithm

To reduce the time of computing the parameter estimates and to increase the chances of reaching a meaningful, and hopefully global, optimum, it is important to provide the optimization algorithm with starting values of the parameters that are as good as possible. By visually inspecting the data we were able to obtain educated guesses of some of the parameters. The same values were used to initiate the parameter estimation for all four data sets. The starting value of M_s_ was set to 3300. Noting that the parameter *k*_4_ determines the relaxation time-scale of X and that the observed re-entry took place at roughly 200 s, we set *k*_4_ = 1/200 = 0.005. If X is considered constant, the initial relaxation time-scale of Mig1 is given by *k*_2_. If we by a very crude visual assessment determine this time-scale to 50 s, we consequently set *k*_2_ = 1/50 = 0.02. The measurement noise appear to be on the scale of a hundred to a few hundreds and its variance, *s*, was set to 40 000. Choosing staring values for the parameters of the random effect covariance matrix is more difficult. We have chosen *ω*_11_ = *ω*_22_ = *ω*_33_ = 0.1 and *ω*_12_ = *ω*_13_ = *ω*_23_ = 0. This roughly corresponds to parameter standard deviations of ± 10% with no covariance between random effect parameters. When all experimental data was used simultaneously for estimation, the experiment-specific parameters inherited the starting values defined above.

### Avoiding a constrained problem

The parameters M_s_, *k*_2_, *k*_4_, and *s* are only meaningful for nonnegative values. To avoid a constrained optimization problem, any strictly positive parameter, *θ*, is transformed according to $\theta =e^{\tilde{\theta }}$, with the new starting value ${\tilde{\theta }}_{s}=\text{log}\;\theta_{s}$. When the parameter estimates have been determined, the values of the transformed parameters must then be transformed back. However, when the parameter uncertainties are determined through the calculation of the Hessian, no parameter transformations are performed. In this case it is not needed since we only evaluate the likelihood function and its gradient for values of the parameters that are known to be positive. As a result, the Hessian and the coefficients of variations derived from it are valid for the original, untransformed parameterization of the model.

### Simple analysis of Mig1 dynamics

A simple analysis was designed to extract the typical values (medians) of the response time, amplitude, and duration of Mig1 dynamics directly from data, without the use of a dynamical model. The amplitude for each cell was defined by maximal difference between the baseline and the subsequent data points, and the response time was defined as the time for the maximizing data point. The duration was defined as the difference in time between the first two data points to in each direction cross the level determined from 50% of the amplitude. The simple analysis was also applied to smoothed and densely resampled data. Smoothed data was generated for each cell by fitting a cubic B-spline to its experimental data, and from this smooth function sampling 1000 data points equidistantly in time.

## Supporting Information

S1 Fig Plots of all individual cell data and model simulations for experiment 1. (PDF)

S2 Fig Plots of all individual cell data and model simulations for experiment 2. (PDF)

S3 Fig Plots of all individual cell data and model simulations for experiment 3. (PDF)

S4 Fig Plots of all individual cell data and model simulations for experiment 4. (PDF)

S5 Fig Plots of all individual cell data and model simulations for experiment 1 when accounting for background fluorescence. (PDF)

S6 Fig Plots of all individual cell data and model simulations for experiment 2 when accounting for background fluorescence. (PDF)

S7 Fig Plots of all individual cell data and model simulations for experiment 3 when accounting for background fluorescence. (PDF)

S8 Fig Plots of all individual cell data and model simulations for experiment 4 when accounting for background fluorescence. (PDF)

S9 Fig Plots of all individual cell data and model simulations for experiment 1 when using all data sets simultaneously. (PDF)

S10 Fig Plots of all individual cell data and model simulations for experiment 2 when using all data sets simultaneously. (PDF)

S11 Fig Plots of all individual cell data and model simulations for experiment 3 when using all data sets simultaneously. (PDF)

S12 Fig Plots of all individual cell data and model simulations for experiment 4 when using all data sets simultaneously. (PDF)

S13 Fig Simple analysis histograms. (PDF)

S1 Table Shrinkage. (PDF)

S2 Table Parameter estimates when accounting for background fluorescence. (PDF)

S3 Table Estimates of random effect covariance and correlation matrices when accounting for background fluorescence. (PDF)

S4 Table Parameter estimates when using all data sets simultaneously. (PDF)

S5 Table Estimates of random effect covariance and correlation matrices when using all data sets simultaneously. (PDF)

S1 Text Parameter estimation when accounting for background fluorescence. (PDF)

S2 Text Parameter estimation when using all data sets simultaneously. (PDF)
