# Correction: A Nonlinear Mixed Effects Approach for Modeling the Cell-To-Cell Variability of Mig1 Dynamics in Yeast

**DOI:** 10.1371/journal.pone.0131657

**Published:** 2015-06-25

**Authors:** The PLOS ONE Staff

## Notice of Republication

This article was republished on June 9^th^, 2015, to correct errors in equations that were introduced during the typesetting process. The publisher apologizes for the errors. Please download this article again to view the correct version. The originally published, uncorrected article and the republished, corrected article are provided here for reference.

## Supporting Information

S1 File Originally published, uncorrected article. (PDF)

S2 File Republished corrected article. (PDF)
