# Supplementary figures and images for: A Nonlinear Mixed Effects Approach for Modeling the Cell-To-Cell Variability of Mig1 Dynamics in Yeast
Source: PLoS One. 2015 Apr 20;10(4):e0124050. doi: 10.1371/journal.pone.0124050 (PMC4404321; doi:10.1371/journal.pone.0124050)

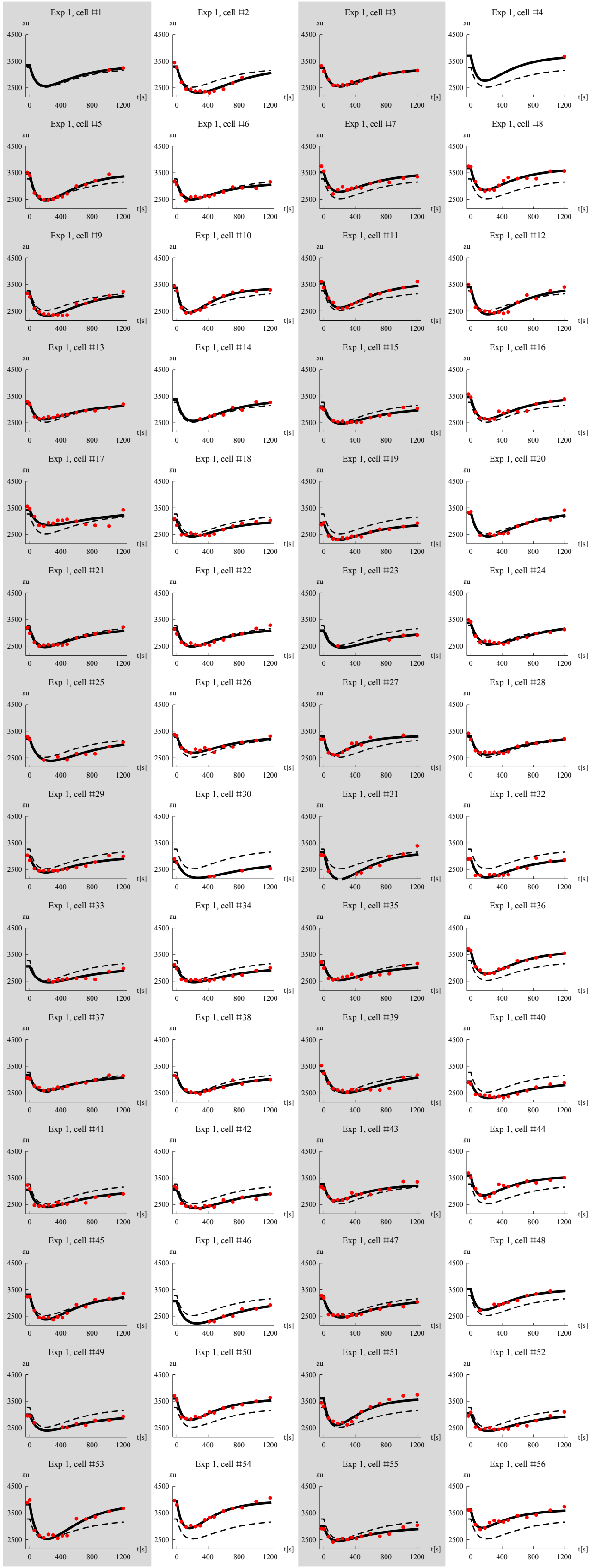

Supplement: S1 Fig — (PDF) [file pone.0124050.s001.pdf]

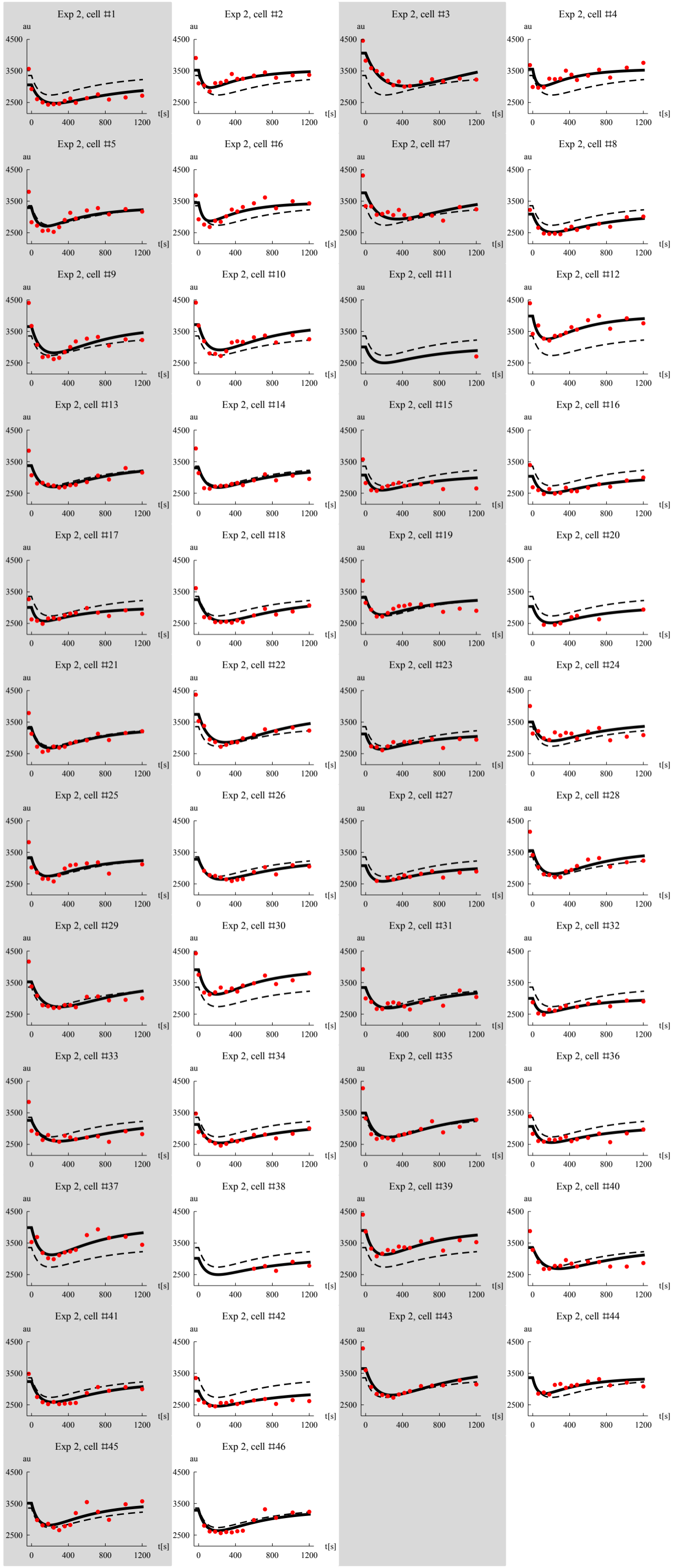

Supplement: S2 Fig — (PDF) [file pone.0124050.s002.pdf]

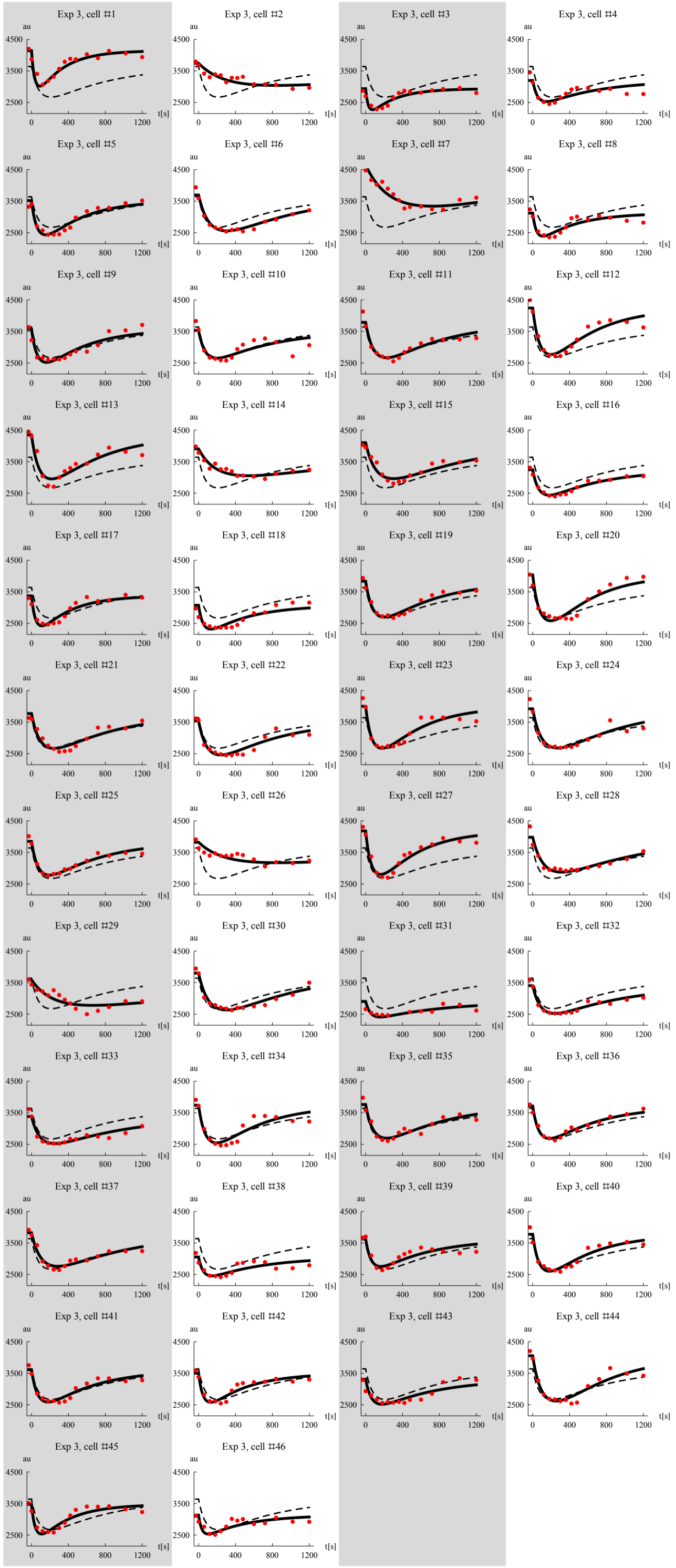

Supplement: S3 Fig — (PDF) [file pone.0124050.s003.pdf]

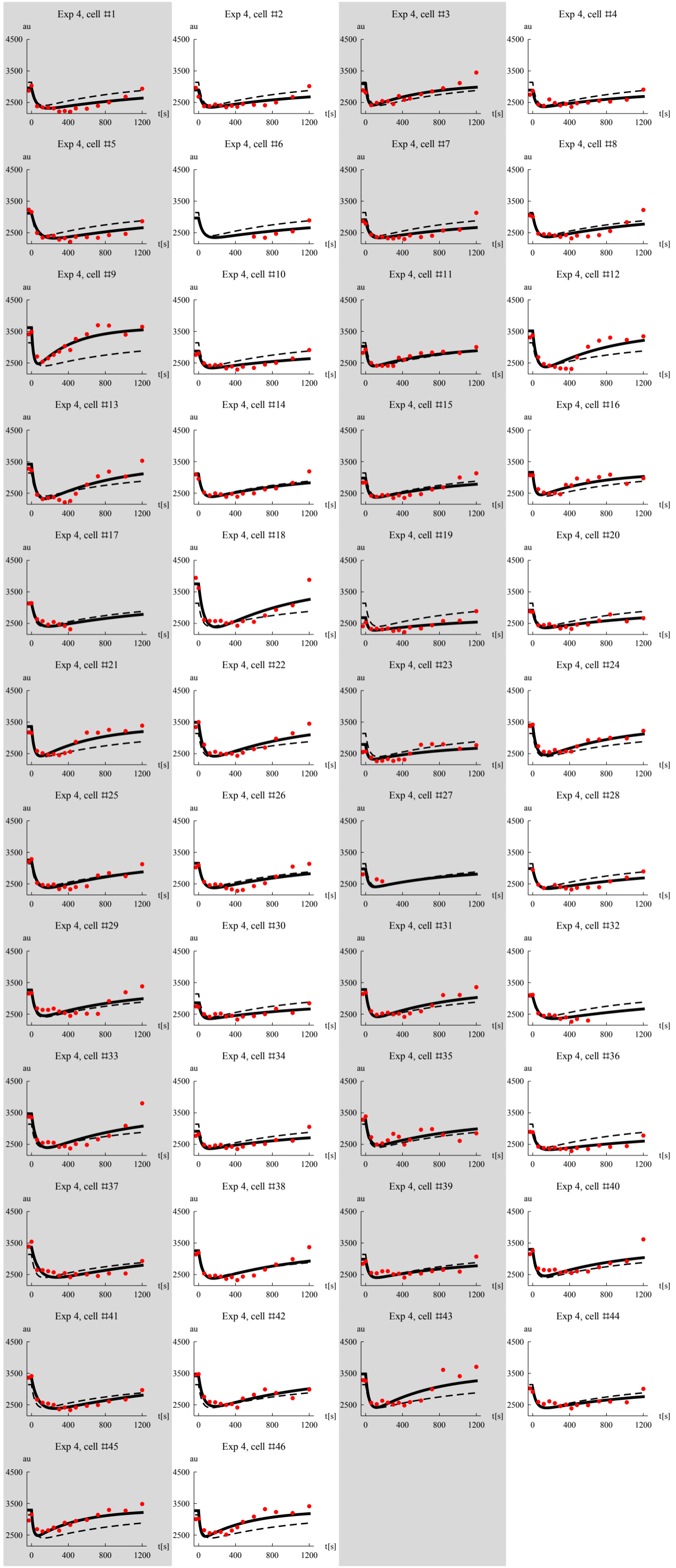

Supplement: S4 Fig — (PDF) [file pone.0124050.s004.pdf]

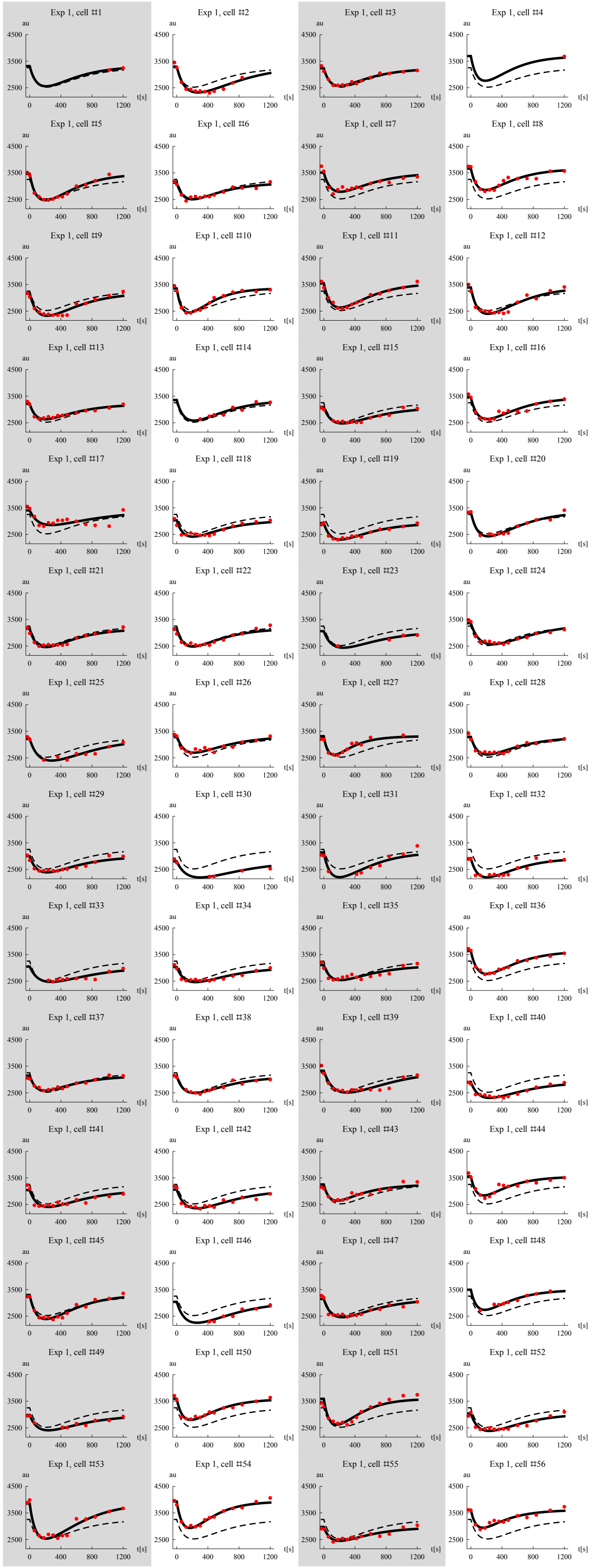

Supplement: S5 Fig — (PDF) [file pone.0124050.s005.pdf]

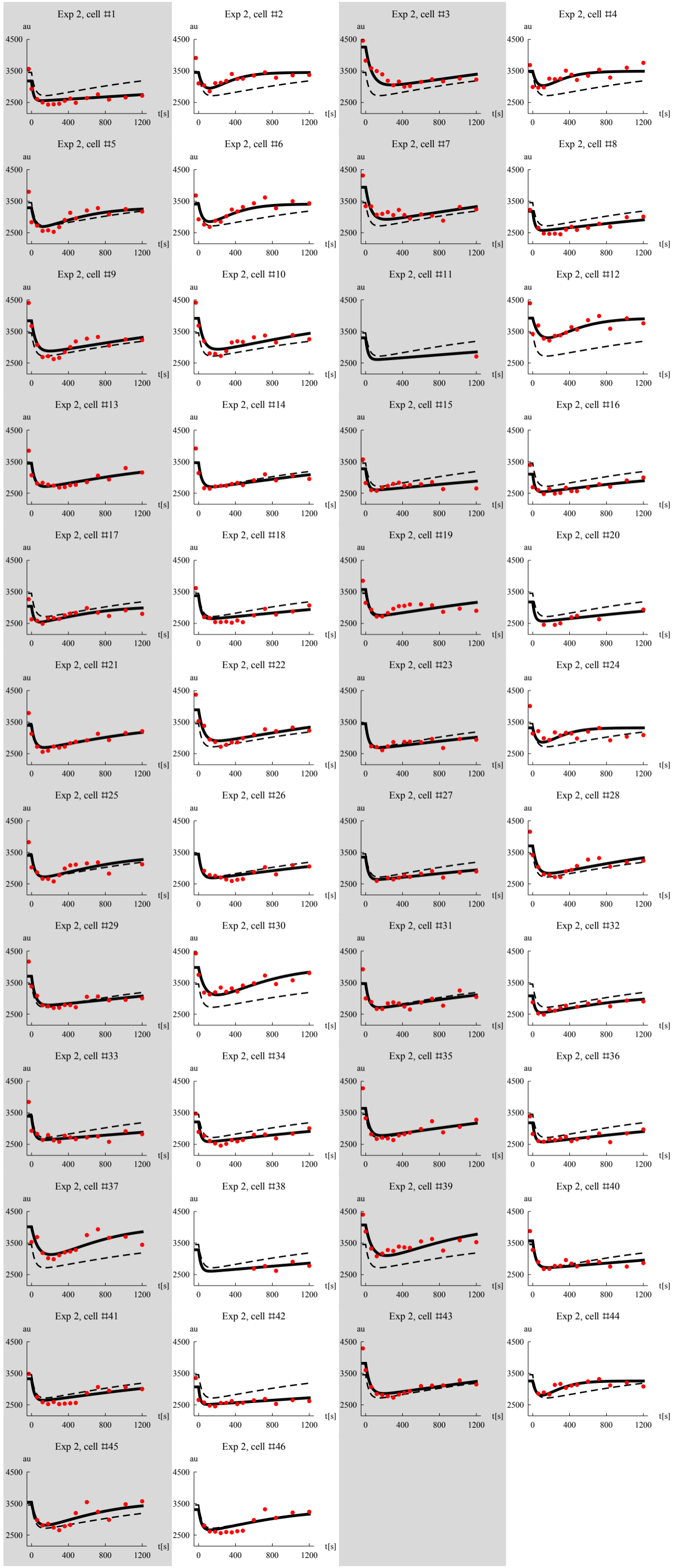

Supplement: S6 Fig — (PDF) [file pone.0124050.s006.pdf]

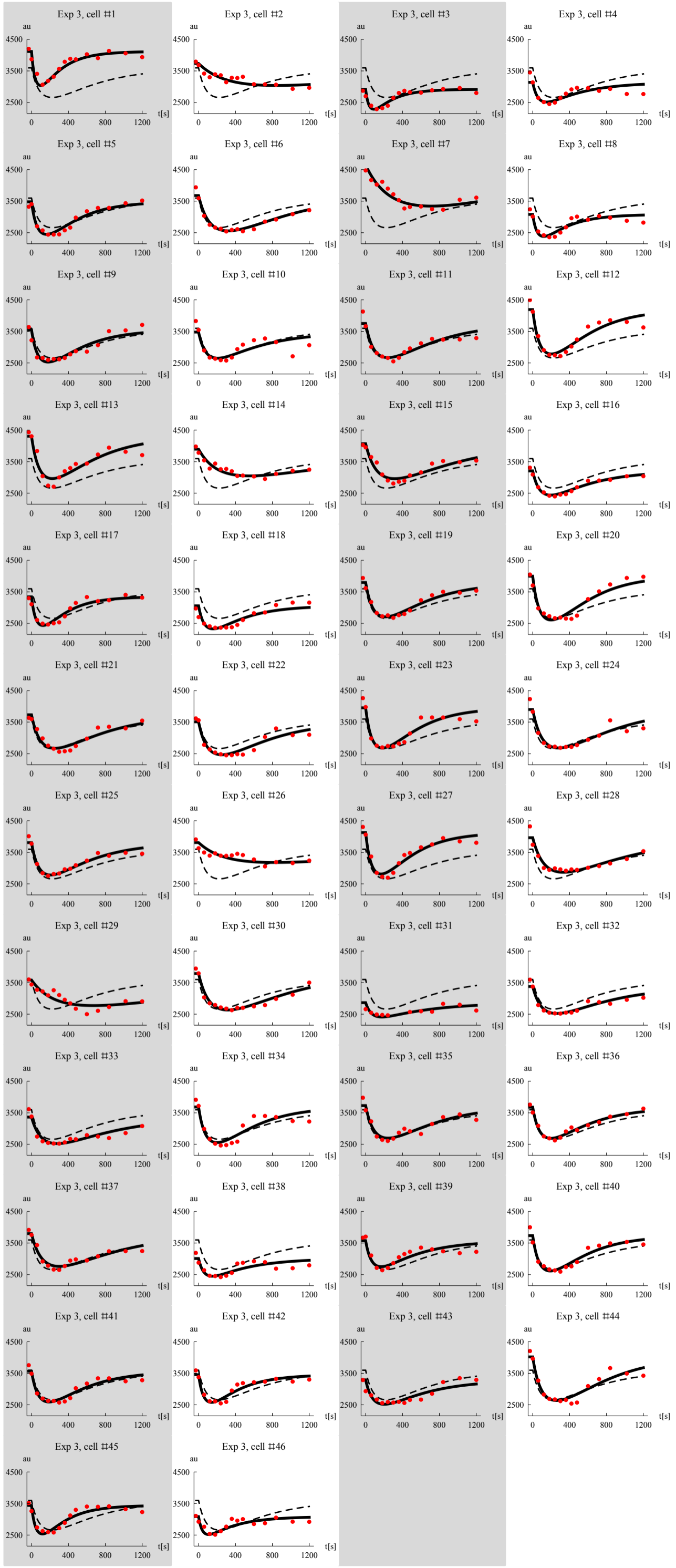

Supplement: S7 Fig — (PDF) [file pone.0124050.s007.pdf]

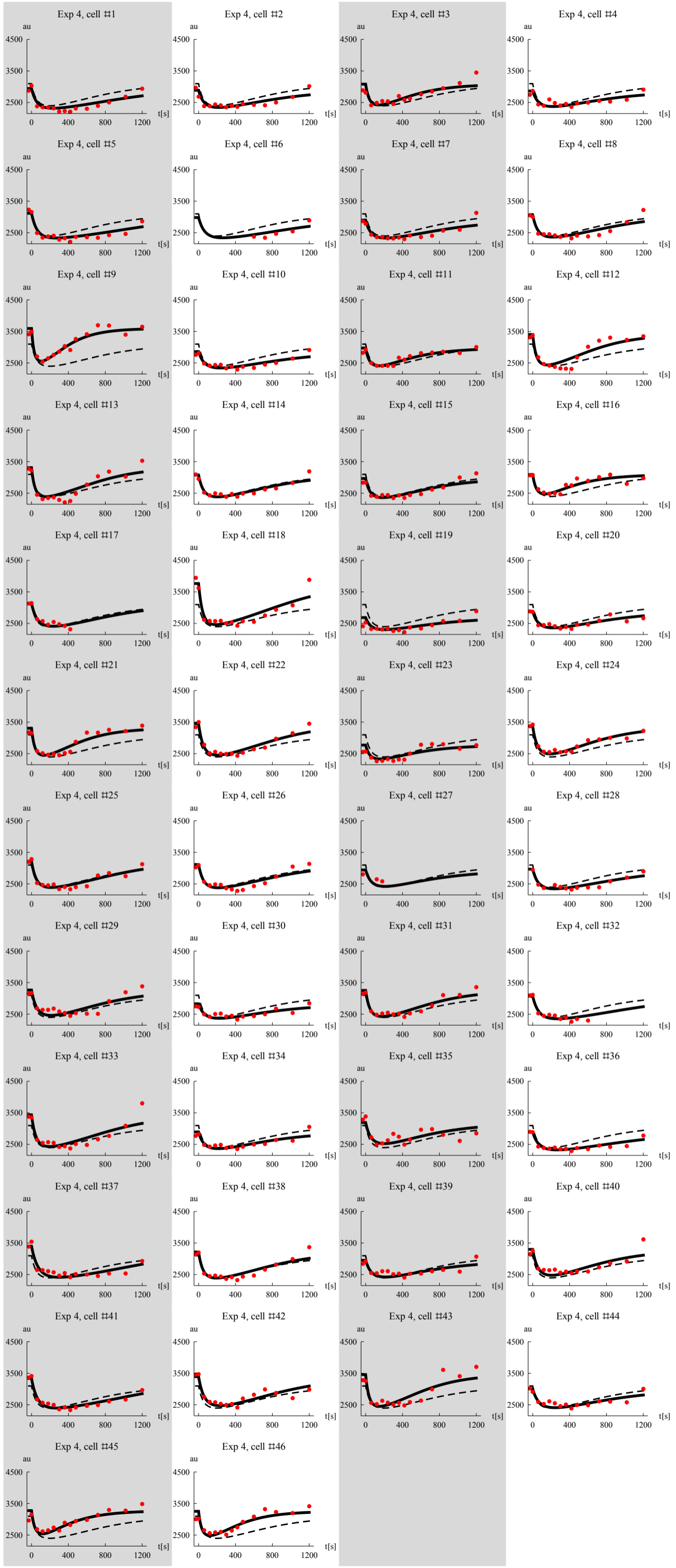

Supplement: S8 Fig — (PDF) [file pone.0124050.s008.pdf]

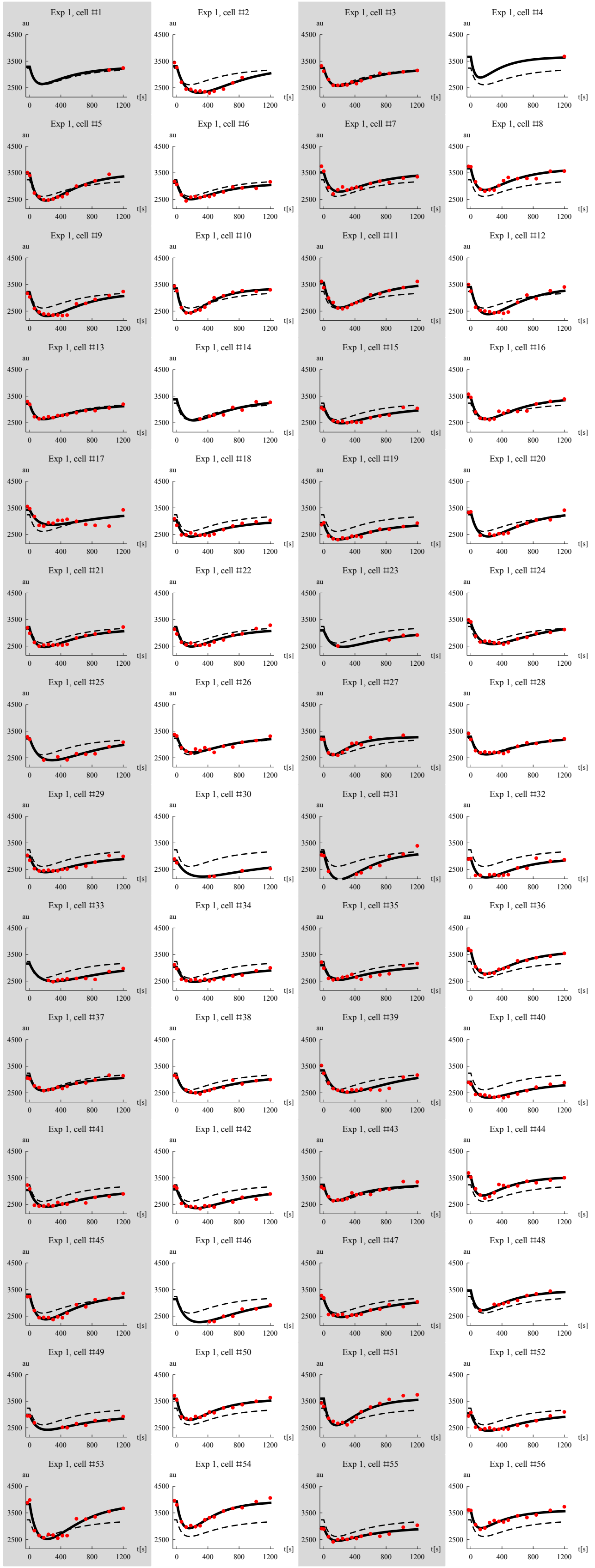

Supplement: S9 Fig — (PDF) [file pone.0124050.s009.pdf]

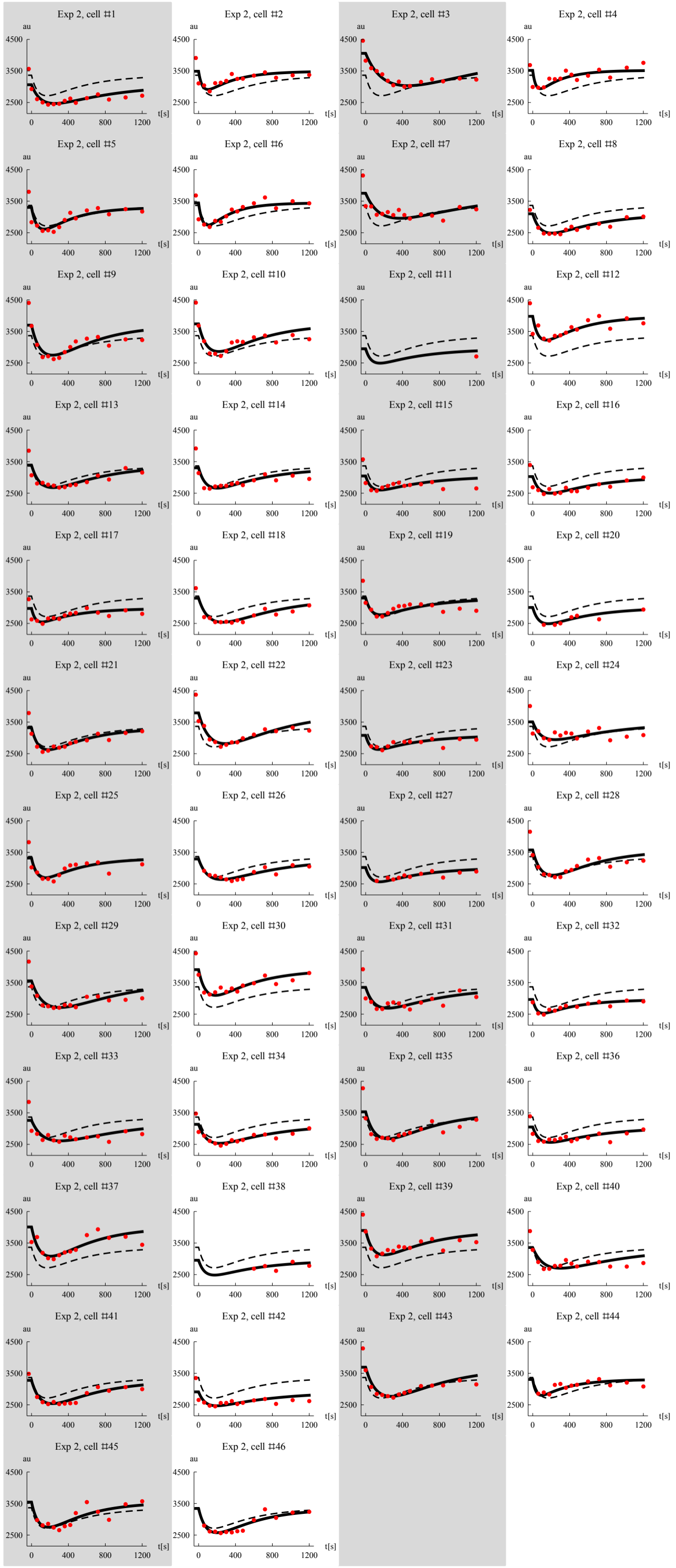

Supplement: S10 Fig — (PDF) [file pone.0124050.s010.pdf]

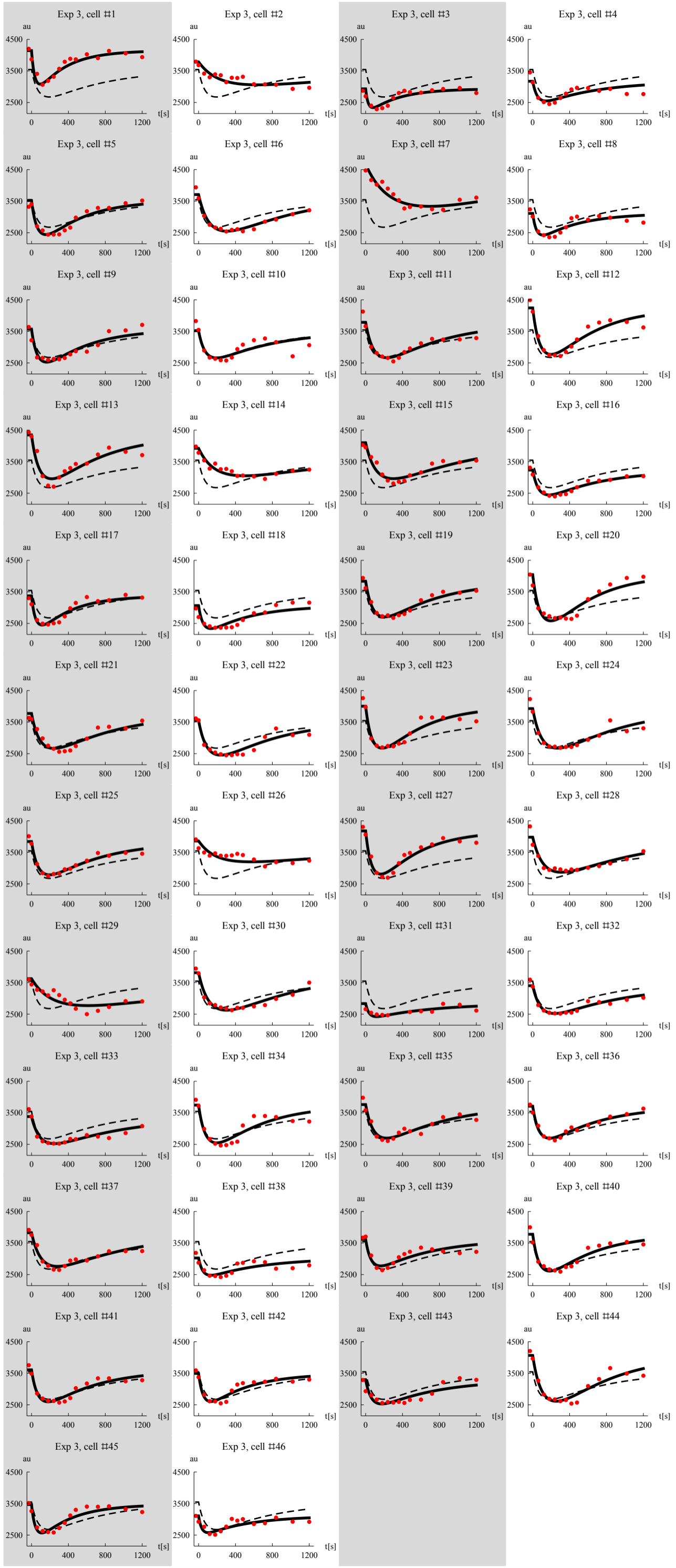

Supplement: S11 Fig — (PDF) [file pone.0124050.s011.pdf]

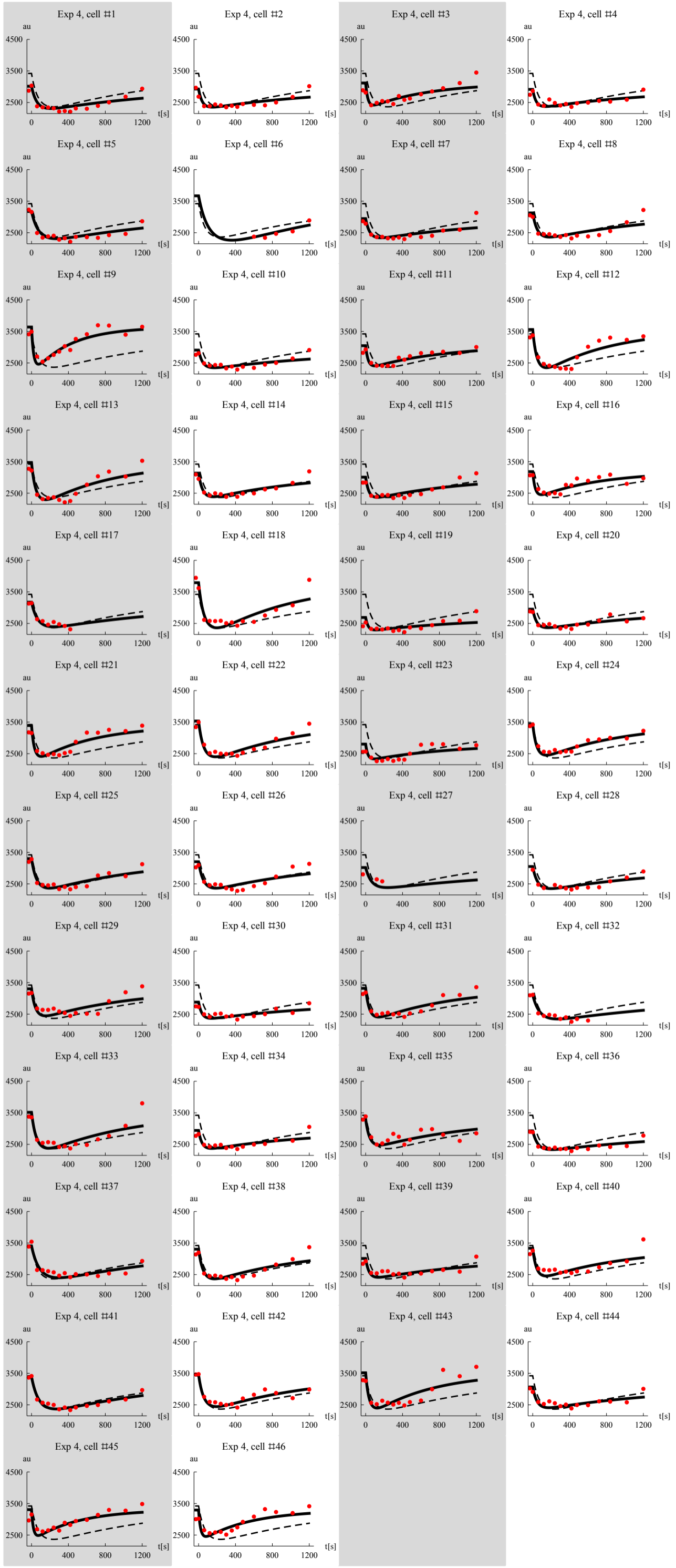

Supplement: S12 Fig — (PDF) [file pone.0124050.s012.pdf]
