# Supplementary material for: A Nonlinear Mixed Effects Approach for Modeling the Cell-To-Cell Variability of Mig1 Dynamics in Yeast
Source: PLoS One. 2015 Apr 20;10(4):e0124050. doi: 10.1371/journal.pone.0124050 (PMC4404321; doi:10.1371/journal.pone.0124050)
Supplement: S13 Fig — (PDF) [file pone.0124050.s013.pdf]

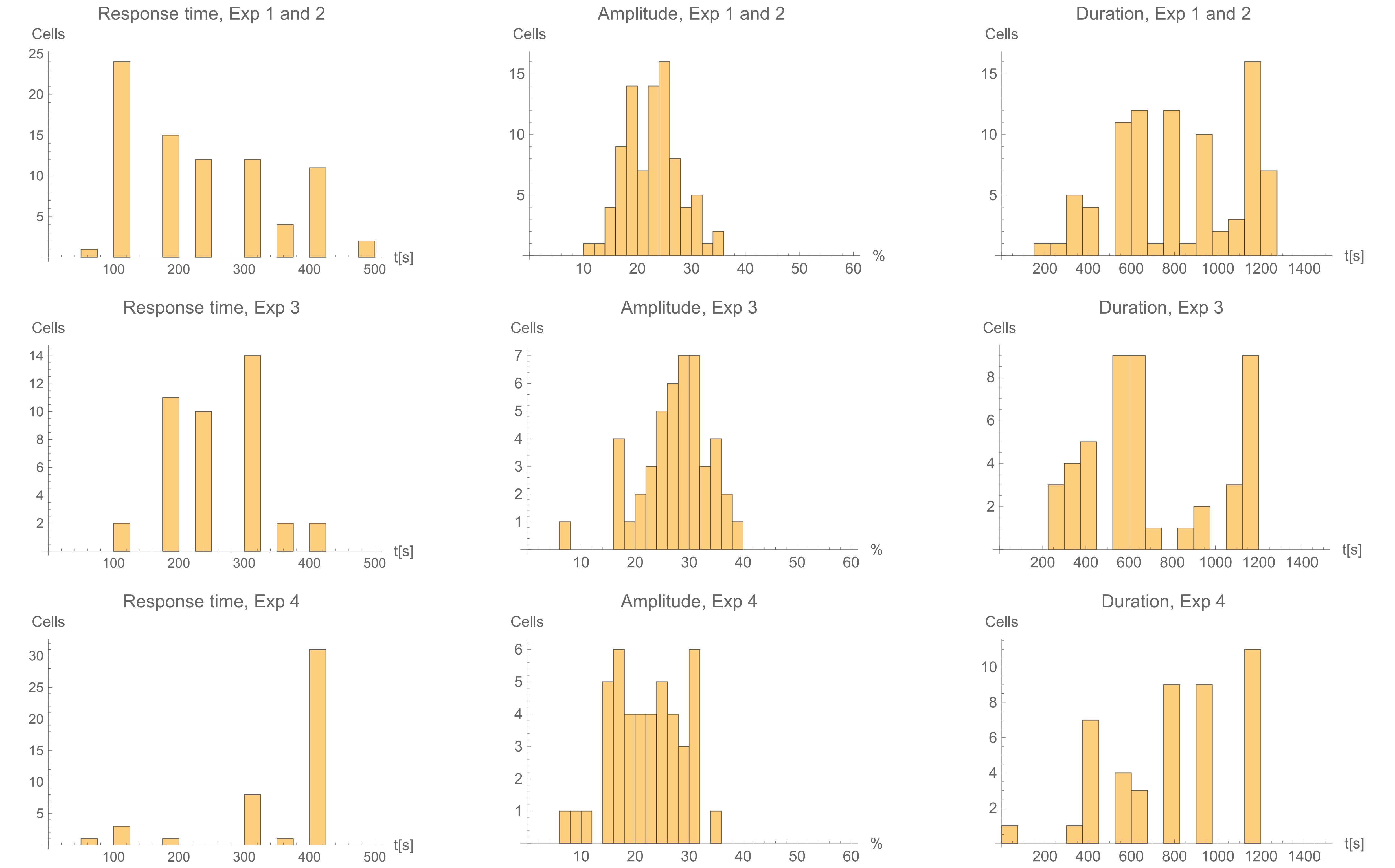
Response time, Exp 1 and 2
Amplitude, Exp 1 and 2
Duration, Exp 1 and 2
Response time, Exp 3
Amplitude, Exp 3
Duration, Exp 3
Response time, Exp 4
Amplitude, Exp 4
Duration, Exp 4
Cells
t[s]
%
