# Supplementary material for: A Nonlinear Mixed Effects Approach for Modeling the Cell-To-Cell Variability of Mig1 Dynamics in Yeast
Source: PLoS One. 2015 Apr 20;10(4):e0124050. doi: 10.1371/journal.pone.0124050 (PMC4404321; doi:10.1371/journal.pone.0124050)
Supplement: S1 Table — (PDF) [file pone.0124050.s014.pdf]

Table 1. Shrinkage

Parameter	Exp 1	Exp 2	Exp 3	Exp 4
η_1	2	4	1	5
η_2	11	26	4	14
η_3	13	20	4	17

η-shrinkage for each of the four experiments separately.
