# Supplementary material for: A Nonlinear Mixed Effects Approach for Modeling the Cell-To-Cell Variability of Mig1 Dynamics in Yeast
Source: PLoS One. 2015 Apr 20;10(4):e0124050. doi: 10.1371/journal.pone.0124050 (PMC4404321; doi:10.1371/journal.pone.0124050)
Supplement: S2 Table — (PDF) [file pone.0124050.s015.pdf]

Parameter estimates for the modified observation model

Parameter	Exp 1	Exp 2	Exp 3	Exp 4
$\overline{\mathrm{M}}_{\mathrm{s}}$	2.35×10^3 (9)	1.3×10^3 (5)	2.45×10^3 (−)	978. (−)
$\overline{k}_2$	0.00751 (10)	0.0263 (16)	0.00798 (−)	0.0183 (−)
$\overline{k}_4$	0.00606 (11)	0.00167 (16)	0.00704 (−)	0.00514 (−)
b	908. (23)	2.15×10^3 (2)	1.15×10^3 (−)	2.12×10^3 (−)
s	8.6×10^3 (6)	34.1×10^3 (6)	20.3×10^3 (−)	18.1×10^3 (−)
ω_{11}	0.0938 (15)	0.0594 (226)	0.11 (−)	0.211 (−)
ω_{12}	0.0479 (33)	−0.227 (20)	0.0587 (−)	0.125 (−)
ω_{13}	0.00517 (322)	−0.0351 (153)	−0.1 (−)	−0.0448 (−)
ω_{22}	0.256 (13)	0.381 (35)	0.354 (−)	0.27 (−)
ω_{23}	−0.00654 (764)	−0.176 (72)	0.486 (−)	0.105 (−)
ω_{33}	0.29 (13)	0.863 (15)	0.535 (−)	0.431 (−)

Estimated parameter values considering each of the four experiments separately, using the observation model including background fluorescence.
