# Supplementary material for: A Nonlinear Mixed Effects Approach for Modeling the Cell-To-Cell Variability of Mig1 Dynamics in Yeast
Source: PLoS One. 2015 Apr 20;10(4):e0124050. doi: 10.1371/journal.pone.0124050 (PMC4404321; doi:10.1371/journal.pone.0124050)
Supplement: S3 Table — (PDF) [file pone.0124050.s016.pdf]

Covariance and correlations matrices for the modified observation model

Exp Nr	$\boldsymbol{\Omega}$	Corr
1	$\begin{pmatrix} 0.011\,(28) & 0.012\,(39) & 0.0015\,(326) \\ & 0.066\,(26) & -0.0019\,(769) \\ & & 0.084\,(26) \end{pmatrix}$	$\begin{pmatrix} 1 & 0.45\,(29) & 0.049\,(317) \\ & 1 & -0.026\,(740) \\ & & 1 \end{pmatrix}$
2	$\begin{pmatrix} 0.056\,(57) & -0.08\,(48) & -0.03\,(160) \\ & 0.18\,(70) & -0.15\,(78) \\ & & 0.75\,(31) \end{pmatrix}$	$\begin{pmatrix} 1 & -0.81\,(26) & -0.15\,(129) \\ & 1 & -0.42\,(65) \\ & & 1 \end{pmatrix}$
3	$\begin{pmatrix} 0.026\,(-) & -0.028\,(-) & -0.053\,(-) \\ & 0.36\,(-) & 0.26\,(-) \\ & & 0.29\,(-) \end{pmatrix}$	$\begin{pmatrix} 1 & -0.29\,(-) & -0.63\,(-) \\ & 1 & 0.81\,(-) \\ & & 1 \end{pmatrix}$
4	$\begin{pmatrix} 0.062\,(-) & 0.029\,(-) & -0.019\,(-) \\ & 0.084\,(-) & 0.045\,(-) \\ & & 0.19\,(-) \end{pmatrix}$	$\begin{pmatrix} 1 & 0.4\,(-) & -0.18\,(-) \\ & 1 & 0.36\,(-) \\ & & 1 \end{pmatrix}$

Covariance and correlations matrices considering each of the four experiments separately, using the observation model including background fluorescence.
