# Supplementary material for: A Nonlinear Mixed Effects Approach for Modeling the Cell-To-Cell Variability of Mig1 Dynamics in Yeast
Source: PLoS One. 2015 Apr 20;10(4):e0124050. doi: 10.1371/journal.pone.0124050 (PMC4404321; doi:10.1371/journal.pone.0124050)
Supplement: S4 Table — (PDF) [file pone.0124050.s017.pdf]

Table 1. Parameter estimates

Parameter	All experiments
$\overline{\mathrm{M}}_{\mathrm{s1}}$	3.24×10^3 (1)
$\overline{\mathrm{M}}_{\mathrm{s2}}$	3.36×10^3 (2)
$\overline{\mathrm{M}}_{\mathrm{s3}}$	3.55×10^3 (1)
$\overline{\mathrm{M}}_{\mathrm{s4}}$	3.42×10^3 (1)
$\bar{k}_2$	0.00605 (4)
$\bar{k}_4$	0.0116 (5)
s_1	8.73×10^3 (6)
s_2	37.1×10^3 (6)
s_3	21.1×10^3 (6)
s_4	24.2×10^3 (6)
ω_{11}	0.0648 (11)
ω_{15}	0.0447 (27)
ω_{16}	0.0155 (127)
ω_{22}	0.0743 (14)
ω_{25}	0.0482 (46)
ω_{26}	−0.0489 (35)
ω_{33}	0.0637 (12)
ω_{35}	0.0447 (24)
ω_{36}	−0.0967 (14)
ω_{44}	0.0235 (30)
ω_{45}	0.0781 (11)
ω_{46}	−0.0561 (20)
ω_{55}	0.299 (7)
ω_{56}	0.3 (11)
ω_{66}	0.565 (7)

Estimated parameter values and their corresponding relative standard error (expressed in percentage in the parenthesis), considering all four experiments simultaneously.
