# Supplementary material for: A Nonlinear Mixed Effects Approach for Modeling the Cell-To-Cell Variability of Mig1 Dynamics in Yeast
Source: PLoS One. 2015 Apr 20;10(4):e0124050. doi: 10.1371/journal.pone.0124050 (PMC4404321; doi:10.1371/journal.pone.0124050)
Supplement: S5 Table — (PDF) [file pone.0124050.s018.pdf]

Table 1. Covariance and correlations matrix

$\boldsymbol{\Omega}$	$\begin{pmatrix} 0.0064\,(25) & 0 & 0 & 0 & 0.018\,(44) & 0.0087\,(128) \\ & 0.01\,(25) & 0 & 0 & -290.\times 10^{-6}\,(3058) & -0.028\,(37) \\ & & 0.015\,(20) & 0 & -0.016\,(36) & -0.055\,(18) \\ & & & 0.0098\,(18) & 0.0065\,(80) & -0.032\,(24) \\ & & & & 0.18\,(13) & 0.17\,(16) \\ & & & & & 0.32\,(13) \end{pmatrix}$
Corr	$\begin{pmatrix} 1 & 0 & 0 & 0 & 0.53\,(34) & 0.19\,(116) \\ & 1 & 0 & 0 & -0.0068\,(2884) & -0.48\,(29) \\ & & 1 & 0 & -0.3\,(32) & -0.78\,(7) \\ & & & 1 & 0.16\,(79) & -0.57\,(15) \\ & & & & 1 & 0.71\,(7) \\ & & & & & 1 \end{pmatrix}$

Covariance and correlations matrices and their corresponding relative standard error (expressed in percentage in the parenthesis), considering all four experiments simultaneously. The random effect parameters described by the first to the sixth row of these matrices are associated with the fixed effect parameters $\overline{\mathrm{M}}_{\mathrm{s1}}$, $\overline{\mathrm{M}}_{\mathrm{s2}}$, $\overline{\mathrm{M}}_{\mathrm{s3}}$, $\overline{\mathrm{M}}_{\mathrm{s4}}$, $\overline{k}_2$, and $\overline{k}_4$, respectively.
