# Supplementary material for: A Nonlinear Mixed Effects Approach for Modeling the Cell-To-Cell Variability of Mig1 Dynamics in Yeast
Source: PLoS One. 2015 Apr 20;10(4):e0124050. doi: 10.1371/journal.pone.0124050 (PMC4404321; doi:10.1371/journal.pone.0124050)
Supplement: S1 Text — (PDF) [file pone.0124050.s019.pdf]

Changing the observation model

Performing parameter estimation with the model accounting for background fluorescence suffered from two kinds of complications: 1) for some starting values the parameter estimation resulted in convergence to local optima that gave substantially worse fits than the original model, and 2) for other starting values the parameter estimation algorithm experienced premature termination in flat regions of the likelihood, possibly close to an optimum but with a negative Hessian that was not positive definite. It is also notable that for some starting values for the parameters, the value of the new parameter b did only change very marginally before the algorithm had terminated. By trying different starting values the optimization algorithm was nevertheless able to a set of parameter values for which the likelihood value was somewhat better than for the original model. This was achieved for all of the four experiments. For experiments 1 and 2, these parameter values could be confirmed as a (local) likelihood optimum with a negative Hessian that was positive definite. However, despite several attempts we were unable to confirm such optima for experiments 3 and 4, and hence the RSDs could not be determined for these estimates. The results of the parameter estimation is shown in Table S1, and the corresponding random effect covariance and correlation matrices are shown in Table S2. Plots of all individual cell data and model simulations for the four different experiments are shown in Figures S5, S6, S7, and S8. Comparing these plots to the corresponding plots from the model without the background fluorescence, they look virtually identical. The fact that the optimization was very sensitive towards the initial parameter values and that two of the estimations terminated with a negative Hessian that was not positive definite indicated that this model had issues both with multiple optima and with practical identifiability. Not knowing the precise shape of this potentially very complex likelihood function, and relying only on asymptotic theory for determining the precision of the estimates, the results from the parameter estimation should be interpreted with caution.
