# Supplementary material for: A Nonlinear Mixed Effects Approach for Modeling the Cell-To-Cell Variability of Mig1 Dynamics in Yeast
Source: PLoS One. 2015 Apr 20;10(4):e0124050. doi: 10.1371/journal.pone.0124050 (PMC4404321; doi:10.1371/journal.pone.0124050)
Supplement: S2 Text — (PDF) [file pone.0124050.s020.pdf]

Using all data sets simultaneously

To account for the fact that the baseline level of the model state variable Mig1(t) may be different in the different experiments, both due to cells being taken from different cell batches and the fact that measured fluorescence intensities may be differently scaled from the actual concentration of Mig1 molecules depending on occasion-specific properties of the experimental setup, the unique parameters $\overline{\mathrm{M}}_{\mathrm{s1}}, \overline{\mathrm{M}}_{\mathrm{s2}}, \overline{\mathrm{M}}_{\mathrm{s3}}$, and $\overline{\mathrm{M}}_{\mathrm{s4}}$, was used for each separate experiment, respectively. The random effect parameter associated with these levels were additionally described by separate parameters, $\eta_{11}, \eta_{12}, \eta_{13}$, and η_{14}. Similarly, the parameter describing the magnitude of the measurement noise, s, was also allowed to take on separate values, s_1, s_2, s_3, and s_4, in each of the four experiments. The fixed effect parameters $\overline{k}_2$ and $\overline{k}_4$, and their associated random effect parameters η_2 and η_3, were on the other hand shared between all experiments. Since there should be no covariance between random effect parameters that are not being used in the same experiments, the matrix $\mathbf{\Omega}$ takes the form

$$\mathbf{\Omega} = \begin{pmatrix} \omega_{11}^2 + \omega_{15}^2 + \omega_{16}^2 & 0 & 0 & 0 & \omega_{15}\omega_{55} + \omega_{16}\omega_{56} & \omega_{16}\omega_{66} \\ 0 & \omega_{22}^2 + \omega_{25}^2 + \omega_{26}^2 & 0 & 0 & \omega_{25}\omega_{55} + \omega_{26}\omega_{56} & \omega_{26}\omega_{66} \\ 0 & 0 & \omega_{33}^2 + \omega_{35}^2 + \omega_{36}^2 & 0 & \omega_{35}\omega_{55} + \omega_{36}\omega_{56} & \omega_{36}\omega_{66} \\ 0 & 0 & 0 & \omega_{44}^2 + \omega_{45}^2 + \omega_{46}^2 & \omega_{45}\omega_{55} + \omega_{46}\omega_{56} & \omega_{46}\omega_{66} \\ \omega_{15}\omega_{55} + \omega_{16}\omega_{56} & \omega_{25}\omega_{55} + \omega_{26}\omega_{56} & \omega_{35}\omega_{55} + \omega_{36}\omega_{56} & \omega_{45}\omega_{55} + \omega_{46}\omega_{56} & \omega_{55}^2 + \omega_{56}^2 & \omega_{56}\omega_{66} \\ \omega_{16}\omega_{66} & \omega_{26}\omega_{66} & \omega_{36}\omega_{66} & \omega_{46}\omega_{66} & \omega_{56}\omega_{66} & \omega_{66}^2 \end{pmatrix},$$

where the total vector of parameters to be estimated is

$$\boldsymbol{\theta} = (\overline{\mathrm{M}}_{\mathrm{s1}}, \overline{\mathrm{M}}_{\mathrm{s2}}, \overline{\mathrm{M}}_{\mathrm{s3}}, \overline{\mathrm{M}}_{\mathrm{s4}}, \overline{k}_2, \overline{k}_4, s_1, s_2, s_3, s_4, \omega_{11}, \omega_{15}, \omega_{16}, \omega_{22}, \omega_{25}, \omega_{26}, \omega_{33}, \omega_{35}, \omega_{36}, \omega_{44}, \omega_{45}, \omega_{46}, \omega_{55}, \omega_{56}, \omega_{66}).$$

The results of the parameter estimation is shown in Table S3 and the corresponding random effect covariance and correlation matrices are shown in Table S4. The experiments-specific parameters have values comparable to those derived from the separate analysis, and the shared parameters were estimated to values being within 10% of the average of the separate estimates. Comparing Table S4 to the separate analysis, we also note that the moderate correlation between the initial Mig1 levels and $\overline{k}_4$ as well as the stronger correlation between $\overline{k}_2$ and $\overline{k}_4$ persists. Shrinkage remained low in the combined analysis, being 10% and 8%, respectively, for η_2 and η_3 Plots of all individual cell data and model simulations for the four different experiments are shown in Figures S9, S10, S11, and S12. These plots show that the simulated single cell Mig1 dynamics, using the $\boldsymbol{\theta}$ and EBEs from the simultaneous analysis, are similar to the corresponding results from the separate analysis.
